# The phylogeny of *Acetobacteraceae*: photosynthetic traits and deranged respiratory enzymes

**DOI:** 10.1128/spectrum.00575-23

**Published:** 2023-11-17

**Authors:** Mauro Degli Esposti, Gabriela Guerrero, Marco A. Rogel, Francisco Issotta, Camila Rojas-Villalobos, Raquel Quatrini, Esperanza Martinez-Romero

**Affiliations:** 1 Center for Genomic Sciences, UNAM Campus de Morelos, Cuernavaca, Morelos, Mexico; 2 Centro Científico y Tecnológico de Excelencia Ciencia & Vida, Fundación Ciencia y Vida, Huechuraba, Santiago, Chile; 3 Departamento de Genética Molecular y Microbiología, Facultad de Ciencias Biológicas, P. Universidad Católica, Santiago, Chile; 4 Facultad de Ingeniería, Arquitectura y Diseño, Universidad San Sebastián, Santiago, Chile; 5 Facultad de Medicina y Ciencia, Universidad San Sebastián, Providencia, Santiago, Chile; Istituto Italiano di Tecnologia, Torino, Piemonte, Italy

**Keywords:** phylogenomics, bacterial phylogeny, energy metabolism, Acetobacteraceae

## Abstract

We present here a comprehensive phylogenomic analysis of *Acetobacteraceae*, a vast group of alphaproteobacteria that has been widely studied for their economic importance. Our results indicate that the ancestor of *Acetobacteraceae* most likely was photosynthetic and evolved via a progressive transition from versatile photoferrotrophy to the incomplete oxidation of organic substrates defining acetous physiology. Vestigial signs of photosynthetic carotenoid metabolism are present in non-photosynthetic acetous taxa that have lost cytochrome oxidase, while their sister taxa retain such traits. The dominant terminal oxidase of acetous bacteria, the *bo*_3_ ubiquinol oxidase, is derived from duplication and diversification of operons present in *Acidocella* taxa that have lost photosynthesis. We analyzed the bioenergetic traits that can compensate for the electron transfer function of photosynthetic reaction centers or constitute alternative pathways for the oxidoreduction of *c*-type cytochromes, such as iron oxidation. The latter pathway bypasses the deranged cytochrome *bc*_1_ complex that is characteristically present in acidophilic taxa due to the loss of conserved ligands in both the Rieske iron-sulfur protein and cytochrome *b* subunit. The deranged or non-functional *bc*_1_ complex may be retained for its structural role in stabilizing Complex I. The combination of our phylogenetic analysis with in-depth functional evaluations indicates that the order Acetobacterales needs to be emended to include three families: *Acetobacteraceae sensu stricto*, *Roseomonadaceae* fam. nov., and *Acidocellaceae* fam. nov.

**IMPORTANCE:**

Acetobacteraceae are one of the best known and most extensively studied groups of bacteria, which nowadays encompasses a variety of taxa that are very different from the vinegar-producing species defining the family. Our paper presents the most detailed phylogeny of all current taxa classified as *Acetobacteraceae*, for which we propose a taxonomic revision. Several of such taxa inhabit some of the most extreme environments on the planet, from the deserts of Antarctica to the Sinai desert, as well as acidic niches in volcanic sites like the one we have been studying in Patagonia. Our work documents the progressive variation of the respiratory chain in early branching Acetobacteraceae into the different respiratory chains of acidophilic taxa such as *Acidocella* and acetous taxa such as *Acetobacter*. Remarkably, several genomes retain remnants of ancestral photosynthetic traits and functional *bc*_1_ complexes. Thus, we propose that the common ancestor of *Acetobacteraceae* was photosynthetic.

## INTRODUCTION

*Acetobacteraceae* constitute a vast subdivision of alphaproteobacteria that requires taxonomic revision, since the family encompasses monophyletic clades comprising multiple genera, most notably the *Roseomonas* clade (1), plus a variety of acidophilic and photosynthetic taxa of uncertain phylogenetic position (2 – 5). Traditionally, *Acetobacteraceae* are divided into acidophilic and acetous groups (2, 4). The acetous group has been studied extensively for the economic relevance of its members in the production of vinegar and other beverages, as well as other biotechnological applications (2, 4, 6). These bacteria share the physiological character of incomplete oxidation of alcohols and sugars under aerobic conditions (2, 4, 6) and phylogenetically form a compact clade that includes symbionts or commensals of insects (7 – 12). Although considered strictly aerobic (2), members of the acetous group can thrive also under the micro-aerobic conditions of insect guts because their genomes contain terminal oxidases with high affinity for oxygen (8, 9, 13 – 15). The evolution of these oxidases, within *Acetobacteraceae* and other alphaproteobacteria, has been studied only partially (9, 13).

Our previous analysis of the evolution of cytochrome *bd* oxidases led to the conclusion that acidophilic members of *Acetobacteraceae*, such as *Acidocella,* contain early branching forms of these oxidases (16). Subsequent phylogenomic analysis of *Acidocella* strains revealed unusual features regarding the other ubiquinol oxidase characteristically present in *Acetobacteraceae*, cytochrome *bo*_3_ (9, 13, 14, 17). This subtype of the A family of heme-copper oxygen reductases (18) likely evolved in acidophilic Fe^2+^-oxidizing bacteria thriving in the same environments where *Acidocell*a taxa generally live (19). It is intriguing that *Acidocella* genomes combine early branching forms of the cytochrome *bd* and *bo*_3_ ubiquinol oxidases with clearly derived features of other components of the respiratory chain (19, 20). These features, and the recent report of a new group of Antarctic *Acetobacteraceae* (21), stimulated an in-depth analysis of the phylogenomics of all *Acetobacteraceae*, expanding previous studies (4, 5, 22, 23) and niches (21, 24).

The energy metabolism of acetous members of *Acetobacteraceae* has been studied in detail (2, 6, 12 – 14). It pivots on terminal oxidases that oxidize ubiquinol and bypass the two cytochrome-containing, proton-pumping complexes that utilize cytochrome *c* as a substrate (9, 13, 14): the *bc*_1_ complex and cytochrome oxidase (COX). Although genes for the *bc*_1_ complex are present in the genomes of several acetous species, their functional relevance is questionable because the same genomes lack COX genes (13). Hence, there is no efficient way of re-oxidizing reduced cytochrome *c* produced by a potentially active *bc*_1_ complex. An equivalent situation is present in *Acidocella* and *Acidiphilum* taxa, which lack conserved histidine residues that are crucial for the structure and function of cytochrome *b*, the central catalytic subunit of the *bc*_1_ complex (Fig. S1). Consequently, acidophilic members of *Acetobacteraceae*, such as *Acidocella,* share a fundamental bioenergetic trait with acetous taxa: a non-functional yet present *bc*_1_ complex. In contrast, *Elioraea*, *Roseomonas,* and *Rhodovastum*, genera that define other clades of *Acetobacteraceae* (1, 2, 4), possess a functional *bc*_1_ complex that not only reduces cytochrome *c* for its subsequent re-oxidation by COX, but also integrates the re-oxidation of ubiquinol produced by the photosynthetic reaction center that is frequently present in these bacteria (Table 1). The *bc*_1_ complex thus becomes the central component of the respiratory chain that functionally distinguishes photosynthetic (and often mildly acidophilic) taxa, such as *Roseomonas* (Table 1) and *Rhodovastum* (25), from truly acidophilic taxa, such as *Acidocella* and *Acetobacter*.

**TABLE 1:** Major characters of Acetobacteraceae taxa encompassing *Elioraea* and the *Roseomonas* clade^*a*^

Taxon	% GC	Mild acidophilic	PufCLM RC	CrtBDEFI carotenoid	Rubisco
*Elioraea thermophila*	70.9		Yes	Yes	Yes
*Elioraea tepidiphila*	71,3		Yes	Yes	
*Elioraea rosea*	69.9		Yes	Yes	
*Elioraea* sp. *Yellowstone*	72.4		Yes	Yes	
*Elioraea tepida*	70.6		Yes	Yes	
*Roseomonas* clade	71.3^*b*^				
*Roseomonas arctica*	69.5		Yes	Yes	
*Roseomonas oryzicola*	71.2		Yes	Yes	
*Falsiroseomonas bella*	71.5	+	Yes	Yes	
*Roseomonas gilardii* sub. *rosea*	70.9	+			
*Roseomonas cervicalis*	69.0				
*Roseomonas deserti*	71.1				
*Belnapia mucosa*	69.8		Yes	Yes	
*Dankookia rubra*	70.1		Yes	Yes	Yes
*Paracraurococcus ruber*	72.8		Yes	Yes	
*Roseicella frigidaeris*	72.5	+	Yes	Yes	Yes
*Roseococcus microcysteis*	70.9		Yes	Yes	
*Rubitrepida flocculans*	73.4		Yes	Yes	
*Sediminicoccus rosea*	73.9		Yes	Yes	
*Crenalkalicoccus roseus*	74.3				
*Caldovatus sediminis*	74.9	+			
*Humitalea rosea*	69.6				
*Rhodovarius lipocyclicus*	69.9	+			
*Siccirubricoccus deserti*	69.8				

^*a*^ The percentage values of GC content were retrieved from the literature [e.g., reference (1)] and verified in the GTDB database Release 07-RS207 (8 April 2022) (https://gtdb.ecogenomic.org/ accessed on 12 October 2022). The character “mild acidophilic” has been considered positive (+) when the pH range of bacterial growth was reported to extend below pH 6. The presence of at least two Puf subunits of the photosynthetic reaction center is indicated by “Yes,” and the same applies to the presence of the major Crt genes for the biosynthesis of carotenoids following the spirilloxanthin pathway (26). The presence of CO_2_-fixing Form I Rubisco (27) is also indicated by “Yes.”

^*b*^ Mean value of the listed taxa of the *Roseomonas* clade, *Roseomonadaceae*.

**Fig 1:**
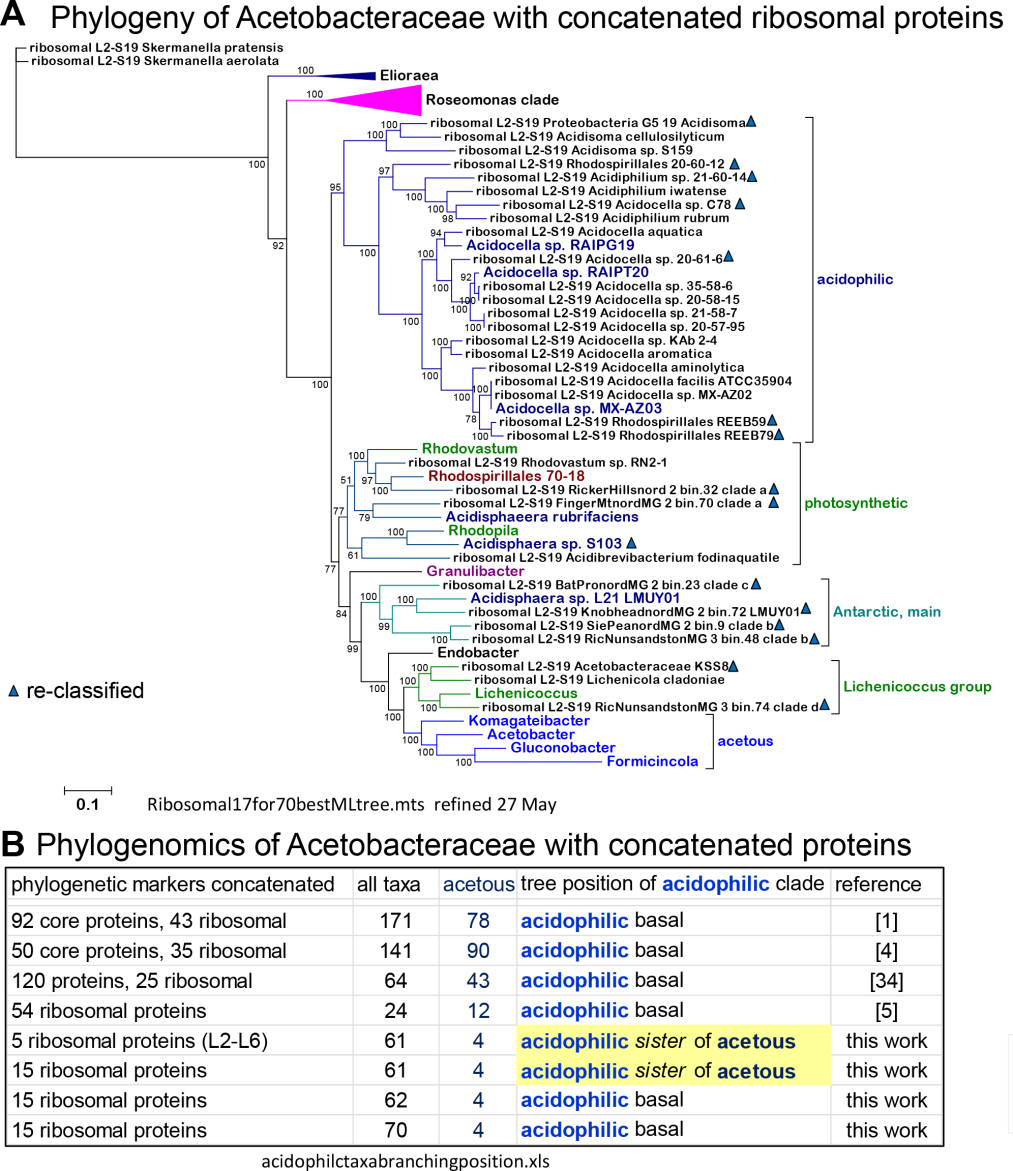
Phylogeny of *Acetobacteraceae* with concatenated alignments of ribosomal proteins. (A) The ML tree was reconstructed with IQ-Tree (38) from a concatenated alignment of 15 ribosomal proteins (L2-6, L10, L14-16, L22, L24, S3, S8, S10, S17, and S19) of 70 taxa encompassing most of those presented in Table 1; Tables S1 and S2. Taxa that had less than 12 complete sequences of the above ribosomal proteins were excluded from the analysis (see Extended Datasheet 2 in the online repository https://osf.io/y6gxt/ for further details). The tree was reconstructed with the best-fit model (39) LG plus gamma = 4, and using an alignment of 2,405 amino acid sites, 40.7% of which were constant. See Fig. S1D for a similar ML tree obtained with an enlarged alignment of 32 concatenated core proteins. The bar quantifies the fractional change per position. The blue triangles indicate taxa re-classified in this study. (B) Summary of the results obtained as in panel A using different taxonomic samplings of *Acetobacteraceae* and also a reduced set of ribosomal proteins (L2-L6 only); these results are compared with those reported in the indicated references, obtained with different combinations of concatenated core proteins including various ribosomal ones. The number of acetous taxa considered in previous studies was much larger than that used in this work, which is focused on other clades of the *Acetobacteraceae* family.

However, the functional separation around the *bc*_1_ complex is difficult to reconcile with the phylogenetic pattern reported for the *Acetobacteraceae* family to date, which shows the acetous clade as a sister to clades including *Rhodovastum* and other photosynthetic taxa, while *Acidocella* and related *Acidiphilium* lie in a distant, basal clade (1–5). Such a branching pattern would imply phylogenetic relatedness for taxa that have clearly different respiratory chains and evolutionary differences between taxa that have fundamentally similar respiratory chains, for example, *Acetobacter* and *Acidocella*. In contrast, phylogenetic trees of cytochrome *b* show a strongly supported sisterhood of the clade including *Acidocella* with the acetous clade of *Acetobacter* (Fig. 1C).

Here, we present a comprehensive survey of the *Acetobacteraceae* phylogeny and relevant functional traits, which rectifies the previous branching order of major clades. We obtained multiple lines of evidence indicating that *Acidocella* and other acidophilic taxa share a common ancestor with the acetous group, contrary to traditional (2) and recent views (3 – 5). We additionally reinforce the re-classification of *Acetobacteraceae*, including *Elioraea,* under the order Acetobacterales (28) and propose two new family subdivisions: *Roseomonadaceae* for the *Roseomonas* clade and *Acidocellaceae* for the acidophilic group. Photosynthesis constitutes a common phenotypic character encompassing Acetobacterales subdivisions up to the acetous clade, which appears to have lost this character concomitantly with the loss of the major COX operon for oxidizing *c*-type cytochromes.

## MATERIALS AND METHODS

### Our approach for a comprehensive analysis of Acetobacteraceae

The objective of this paper was to produce a comprehensive analysis of the *Acetobacteraceae* family encompassing both the taxonomic breath and the functional diversity in the energy metabolism of the family. To accomplish this objective, we initially relied on the DSMZ repository [cf. reference (4)], which listed 61 child genera of *Acetobacteraceae* (https://lpsn.dsmz.de/family/acetobacteraceae, first accessed on 26 May 2022). We then examined the GDTB database (28) (https://gtdb.ecogenomic.org/, first accessed on 5 September 2022), which included a total of 77 genera. Several of these genera did not correspond to those present in the DSMZ repository because they were based upon metagenome-assembled genomes (MAGs) or non-validated taxa (28), for which little genomic information was retrievable from publicly available NCBI resources. Useful genomic information was available for a set of taxa that incompletely overlapped those present in either the GTDB database or the DMSZ repository. We generally used strong taxon density, namely, wide sets of taxa, to evaluate the phylogenetic space of acidophilic taxa to which *Acidocella* belongs. Except for members of *Acidocella* and sister genera, we endeavored to limit taxonomic redundancy in terms of either protein similarity or taxonomic relatedness in the phylogenetic analysis of marker proteins. Along this effort, we analyzed many Antarctic MAGs (21) and novel MAGs contributed by this study. All the taxa studied and their genomic characteristics are listed in Extended Datasheet 1 in the repository https://osf.io/y6gxt/, which is associated with this manuscript.

### Bacterial strains and genomic analysis

We report here the novel genome of a strain derived from the original glycerol storage in which we maintained previously reported *Acidocella* MX-AZ02 (17); we re-isolated the strain from glycerol and selected one colony, which turned out to be a different strain than the original and was thus named MX-AZ03. Its DNA was extracted with the DNA Isolation Kit for Cells and Tissue (Roche), and genomic sequences were obtained using the PacBio RSII single-molecule real-time protocol RS_HGAP_Assembly.3 V2.2.0 from the SMRT portal version 2.3.0.140893 from Pacific Biosciences (Menlo Park, CA, USA). We used the following default parameters: minimum subread length = 500; minimum polymerase read quality = 0.80; and minimum polymerase read length = 100. The program “Flye assembler” version 2.8.3-b1695 (29) was then used to obtain a closed replicon. The genomic characteristics of MX-AZ03 obtained with CheckM (30) are presented in Table S1 and in Extended Datasheet 1 of the repository https://osf.io/y6gxt/.

### Metagenomic analysis

Environmental samples for metagenomic sequencing were obtained from the water column at two sampling points along Rio Agrio Inferior (Neuquén province, Patagonia, Argentina), designated RAI-PG (−37,82884 S; −70,96715 W; 1,516 m.a.s.l.; average temperature 16.7ºC; average pH 3.2) and RAI-PT (−37,81318 S; −70,85157 W; 1,348 m.a.s.l.; average temperature 15.8ºC; average pH 4.4). Cells were separated from larger debris and sediment particles by serial filtration with 0.45- and 0.22-µm filters and preserved at −80°C. Prior to DNA extraction, the frozen filters were sheared, and the resulting pieces were placed in Eppendorf tubes, to which 1 mL of Tris-EDTA buffer was added. After vortexing, the suspension was processed for DNA extraction using phenol-chloroform-isoamyl alcohol. The DNA obtained was purified using the Genomic DNA Clean & Concentrator Kit (Zymo), quantified by fluorescence using the Quant-iT PicoGreen dsDNA Kit (Thermo Fisher), and qualitatively verified by spectrophotometry. Nearly 20 million paired-end (PE) reads were generated through Illumina sequencing systems PE 150 pb on the Hiseq platform through the CD Genomics sequencing service (https://www.cd-genomics.com, NY, USA). A sequence quality check was performed using fastqc v0.11.5 (Babraham Bioinformatics), while adapter removal and trimming were carried out with fastp v0.23.1. Shotgun reads with a >Q35 quality score were retained and *de novo* assembled using SPAdes v3.15.2 built in the pipeline SqueezeMeta v1.5.1 (31) with the parameters: -m sequential -t 90 -a spades -assembly_options ---meta --only-assembler y -t 90¨. Binning was performed using MaxBin2 v2.2.7 (32), Metabat2 (33), and CONCOCT v1.1.0 (25). Genomic characteristics were evaluated using CheckM (30), while preliminary taxonomic assignments were undertaken with GTDB-TK v2.1.0 (28). Additional methodological details are presented in the Extended Methods document posted in the repository https://osf.io/y6gxt/.

### Functional annotation of genes, proteins, and genomes

The analysis for gene annotation and function was initially performed on metagenomic data using conventional methods (19), exploiting multiple databases: GenBank, eggNOG KEGG, and Pfam (June 2022 versions). Details for the functional annotation and specific systems used are listed in Extended Datasheet 2 posted in the repository https://osf.io/y6gxt/, which is associated with the manuscript. Subsequently, genes encoding bioenergetic proteins were analyzed by iterative PSI-BLAST searches to evaluate the completeness of the conserved signatures defining function and the genomic clusters characteristic of complete operons, such as those of COX, essentially as described previously (34, 35).

### Phylogenetic analysis and taxonomic classification

**Fig 2:**
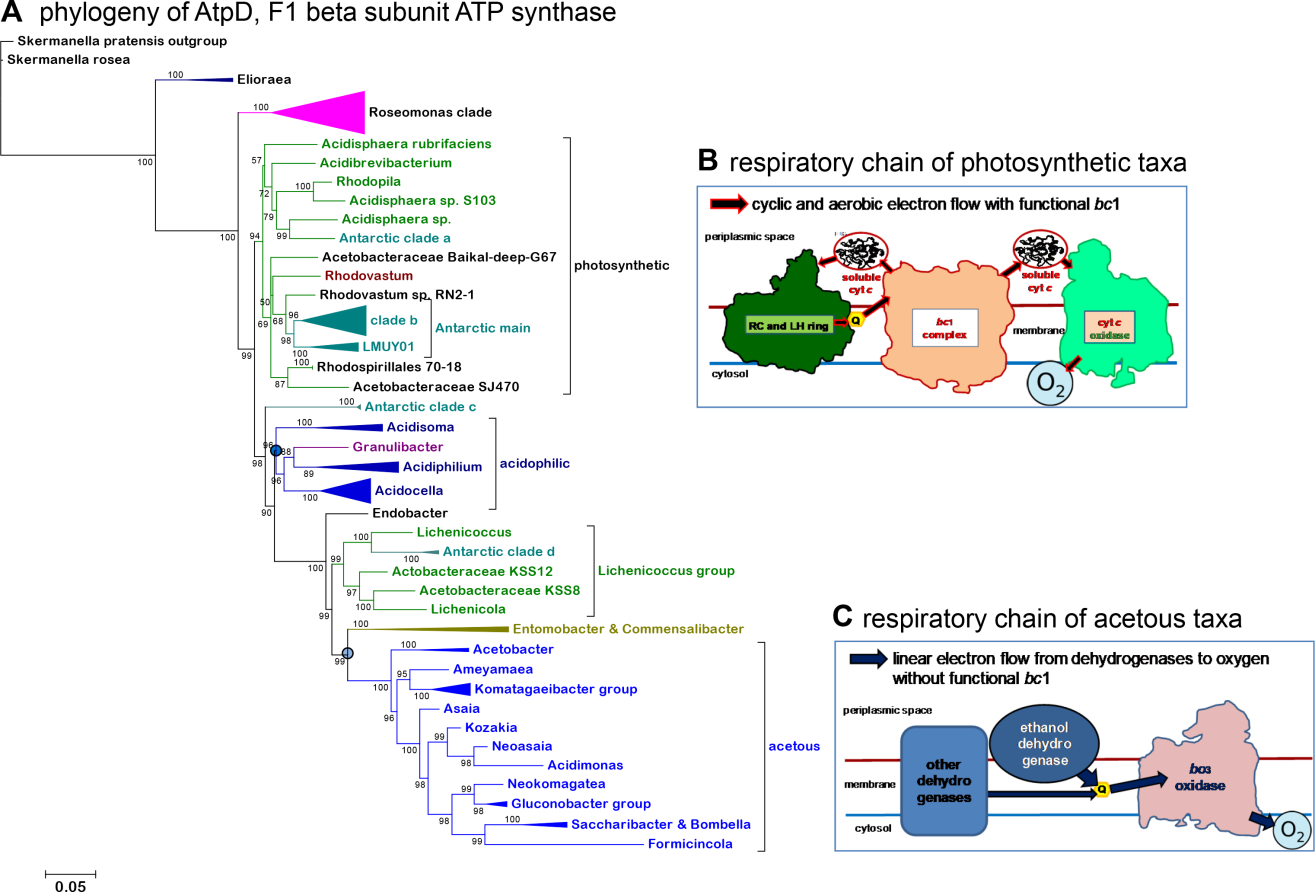
Phylogenetic profile of *Acetobacteraceae* with different conserved markers. (**A)** An alignment of AtpD, the beta subunit of the F1F0 ATP synthase, was first built using ClustalW and then refined manually as described earlier (34, 35) to reconstruct the ML tree with the IQ-Tree program and the best-fit model LG and gamma = 4 (39). The alignment had 122 sequences, including those representing various clades of Antarctic MAGs (Table 2) and most *Acidocella* MAGs listed in Table S1, with a total of 498 amino acid sites, 49.8% of which were constant. The bar quantifies the fractional change per position as in Fig. 1A. Note that the position of *Granulibacter* is shifted from its most common placement at the base of the acetous clade (cf. Fig. 1A), most likely due to the highly divergent nature of its AtpD protein. The blue round symbols indicate the nodes for the sister acidophilic and acetous clades plus their subtending taxa. (**B)** Simplified scheme for the aerobic respiratory chain of *Rhodovastum,* which is representative of other photosynthetic taxa of *Acetobacteraceae*, including those of the *Elioraea* genus and the *Roseomonas* clade. (**C)** Simplified scheme for the respiratory chain of acetous taxa, in which the *bc*_1_ complex, when present, is fundamentally inactive (see text).

Protein sequences retrieved from BLAST searches and various MAGs used in this study were first aligned using ClustalW embedded into MEGA programs and then manually refined using standard procedures that have been reported earlier (34, 35). N- and C-terminal regions were rarely trimmed to preserve information; indeed, bioenergetic proteins such as NuoD and the Rieske iron-sulfur protein (ISP) subunit of the *bc*_1_ complex have very conserved C-terminal regions. Phylogenetic trees were initially reconstructed using the neighbor joining (NJ) approach using the MEGA5 program (usually with the JTT model and 500 bootstraps) to orient the phylogenetic analysis. Subsequently, robust phylogenies were obtained in most cases with maximum likelihood (ML) inference (19, 34 – 36) using the program IQ-Tree as described recently (34) and methodological details that are presented in the figure legend. In some cases, Bayesian inference was additionally used (35). Following earlier works (37), we utilized both concatenated alignments of ribosomal proteins and single markers with strong phylogenetic signals, including the 16S rRNA gene (Fig. S1A) and the beta subunit of the F1 part of rotor-stator ATP synthase, called AtpD here after its gene *atpD* (as in Fig. 2A). We chose this protein marker because of its ubiquity and strong conservation across *Acetobacteraceae* (over 50%, hence very similar to the degree of conservation of 16S rRNA sequences). Moreover, the hydrophilic character of AtpD attenuates possible artifacts deriving from the substantially different GC content among diverse groups of *Acetobacteraceae* (4), since such artifacts are more pronounced for hydrophobic residues. Indeed, phylogenetic trees obtained with AtpD show stronger and better resolved internal nodes than those obtained with the very hydrophobic NuoL subunit of Complex I, despite the longer sequence of the latter (34). Trees similar to those of AtpD were reconstructed with the NuoD subunit of Complex I, which is hardly affected by artifacts derived from compositional and other biases (34). NuoD is significantly less conserved than AtpD across *Acetobacteraceae*, and its increased sequence variation provides additional characters to resolve the placement of “difficult” taxa such as *Granulibacter*, which has divergent proteins forming long branches. In principle, concatenated alignments of ribosomal proteins may provide better phylogenetic patterns than alignments of single marker proteins. However, we found that the taxonomic frequency of individual ribosomal proteins is not homogeneous across *Acetobacteraceae* (see Extended Datasheet 2 posted in the repository https://osf.io/y6gxt/ for details). We assembled a set of 15 ribosomal proteins that were present in the majority of these MAGs and in representative genomes of the rest of the family (using a threshold of three missing sequences for each protein) (Extended Datasheet 2 in the repository https://osf.io/y6gxt/ associated with this paper) to generate phylogenetic trees such as that shown in Fig. 1A. We noted that the branching pattern of such phylogenetic trees showed instability in their central region, despite the apparent strong support for various nodes. This was especially relevant for defining the sister clade to the acetous clade (Fig. 1B). Conversely, the tips of the branches consistently contained the same taxa in trees reconstructed with either concatenated ribosomal proteins or single marker proteins, thereby providing robust information for the taxonomic classification of the MAGs reported here. The same principle was applied for re-classifying previously reported MAGs that were either considered Rhodospirillales bacteria or misclassified, as in the case of *Acidocella* sp. C78, which firmly clusters with *Acidiphilium* taxa (Fig. 1A and 2A). Additional information was used to define the major clades of Antarctic MAGs (21): phylogenetic analyses with additional bioenergetic proteins such as cytochrome *b* (Fig. S1C), consideration of the length of marker proteins, and presence of photosynthetic traits (Table 2). We generally used proteins or genes from *Skermanella* to provide a valid root to our trees, contrary to previous questionable choices (1, 4, 5); *Skermanella* is a photosynthetic genus of the family *Azospirillaceae*, from which *Acetobacteraceae* branched off (3, 37).

**TABLE 2:** Phylogenetic and trait characteristics of Antarctic MAGs^*a*^

MAGs	Clade	Genome	nSor	PS	AtpD (aa)	NuoD (aa)	COX3 (aa)
**RickerHillsnord_2_bin.32**	**a - 70-18**	Medium	-		-	410	301
RicNunsandstonMG_3_bin.94	**a - 70-18**	HQ	1		476	402	289
RicNunsandstonMG_3_bin.43	a′	MQ	2	1	476	416	295
PudButnordMG_2_bin.3	a′	HQ	2		476	416	291
RicNunsandstonMG_3_bin.70	a′	C			480	-	259
**FingerMtnordMG_2_bin.70**	*Rhodopila*	HQ	1	1	476	408	288
**SiePeanordMG_2_bin.9**	b	HQ	1	1	476	417	291
**RicNunsandstonMG_3_bin.48**	b	HQ	2	2	507,476	417	295
SiePeanordMG_2_bin.15	b	HQ	2	1	476	417	295
PudButnordMG_2_bin.90	b	HQ	2.5	2	476	417	291
UniValsudMG_2_bin.112	b	HQ	2	1	476	417	295
RicNunsandstonMG_4_bin.69	b	HQ	1	1	477,469	417	293
LinTernordMG_2_bin.21	b	HQ	1	2	476	417	293
RickerHillsnord_2_bin.54	b	HQ	1	1	477	417	293
RicNunsandstonMG_3_bin.78	b	HQ	2		476,487	417	298
FingerMtnordMG_2_bin.121	b	MQ	1	1	477	-	293
SiePeasudMG_2_bin.54	b	MQ	1	1	525,476	417	291
PudButsudMG_2_bin.32	b	MQ	3 or more	1	476	-	291
PudButsudMG_2_bin.57	b	MQ	2	1	476	417	-
UniValnordMG_2_bin.46	b	MQ	1		476	417	293
FingerMtnordMG_2_bin.53	b	C		Partial	476	-	290
RickerHillsnord_2_bin.38	b	C	2	1	-	417	291
**KnobheadnordMG_2_bin.72**	LMUY01	HQ		1	475,470	412	281
UniValnordMG_2_bin.66	LMUY01	HQ	2		476	406	284
MtNewZealnordMG_2_bin.13	LMUY01	HQ	2		476	406	283
**RicNunsandstonMG_4_bin.87**	LMUY01	HQ	3		476	406	283
RicNunsandstonMG_3_bin.69	LMUY01	HQ	2		475	412	281
MtNewZealnordMG_2_bin.25	LMUY01	HQ	2		475	412	281
FingerMtnordMG_2_bin.15	LMUY01	C	2		476	406	288
**BatPronordMG_2_bin.23**	c	HQ	2		480	415	281
SiePeanordMG_2_bin.20	c	HQ	2		480	319	283
UniValnordMG_2_bin.23	c	HQ	1		480	415	283
RicNunsandstonMG_4_bin.37	c	HQ	1		480	415	283
RickerHillsnord_2_bin.48	c	HQ	1		480	415	283
KnobheadnordMG_2_bin.22	c	HQ	2		480	415	A2 type
KnobheadnordMG_2_bin.10	c	HQ	1		480	415	A2 type
TrioNunataknord_2_bin.115	c	HQ	1		480	415	A2 type
FingerMtsudMG_2_bin.49	c	HQ	1		480	415	-
**RicNunsandstonMG_3_bin.74**	d *Lichenicoccus*	HQ	2		500	416	284
**FingerMtnordMG_2_bin.57**	d *Lichenicoccus*	HQ	2		493	416,417	283
**SiePeasudMG_2_bin.35**	*Siccirubricoccus*	HQ	3		475	418	289

^*a*^ The table lists 41 Antarctic MAGs classified among Acetobacterales with medium- to high-quality genomes (21). The amino acid lengths of the three proteins defining key traits discussed in this work are shown because they provide additional information about the various clades, since they tend to have nearly identical numbers of amino acids (aa) within each clade. PS indicates a strong photosynthetic character for the concomitant presence of *Puf*, *Crt*, and *Chl* genes for RC, carotenoid, and chlorophyll biosynthesis, generally clustered together in the genome (26, 40). Some members of clade b appear to have two of such photosynthetic gene clusters. Abbreviations: HQ, high-quality genome (21); MQ, medium-quality genome (21); C, genome contaminations exceeding 5%; nSor, novel Sulfoxide-Q oxidoreductase. See Extended Datasheet 1 in the repository https://osf.io/y6gxt/ for further genomic details.

## RESULTS AND DISCUSSION

### General features of the *Acetobacteraceae* phylogeny

In the course of this study, we realized that some MAGs classified as Rhodospirillales and Proteobacteria could also be part of the *Acetobacteraceae* family since their proteins clustered within the clade of orthologs from *Acidocella* or other genera of acidophilic *Acetobacteraceae*. Because such MAGs clearly required re-classification, we needed to integrate genomic information available from different resources (listed in Extended Datasheet 1 posted in the online repository https://osf.io/y6gxt/ associated with the manuscript) with our own phylogenetic results to comprehensively analyze *Acetobacteraceae* taxa. This approach of integrated analysis does not conform to standard bioinformatic studies that usually start with a set of taxa retrieved from a single resource. If we had applied standardized selection criteria, we would not have obtained a truly comprehensive analysis of *Acetobacteraceae*, as presented in Table S1. This table focuses on the genus *Acidocella*, to which we contribute additional taxa here. Moreover, our integrated approach enabled flexibility for functional genomic analysis; we progressively expanded or adjusted the taxonomic sampling of *Acetobacteracae* and outgroup taxa depending upon the presence of trait-defining proteins.

To frame the evolutionary steps that originated the diverse bioenergetic traits of *Acetobacteraceae* (Fig. 2B and C), we undertook a comprehensive analysis of the phylogeny of the entire family using concatenated core proteins and different single-copy markers, comparing the resulting trees with those recently reported in the literature. The family *Acetobacteraceae*, recently proposed to form the single family of the order Acetobacterales (28), is dominated by the acetous group (2) in both the overall number of taxa and the proportion of validated genera [ca. 50% of all genera in reference (4)]. Intriguingly, all acetous taxa encompass a compact phylogenetic space that begins with either endophytic *Endobacter* (41) or pathogenic *Granulibacter* and ends with late-branching symbionts of bees and ants, *Bombella* and *Formicincola* [Fig. 1A and 2A; Fig. S1A and D; cf. references (1, 2, 4, 5, 36, 41)]. This compact phylogenetic pattern implies that the fundamental contours of the acetous clade can be defined by a few representatives of the major subclades with acetous character, for example, *Acetobacter* and *Gluconobacter*, as shown in Fig. 1A. *Granulibacter* appears to have an odd position in phylogenetic trees obtained with the single marker AtpD (Fig. 2A), chiefly due to the highly divergent nature of its homolog protein.

**Fig 3:**
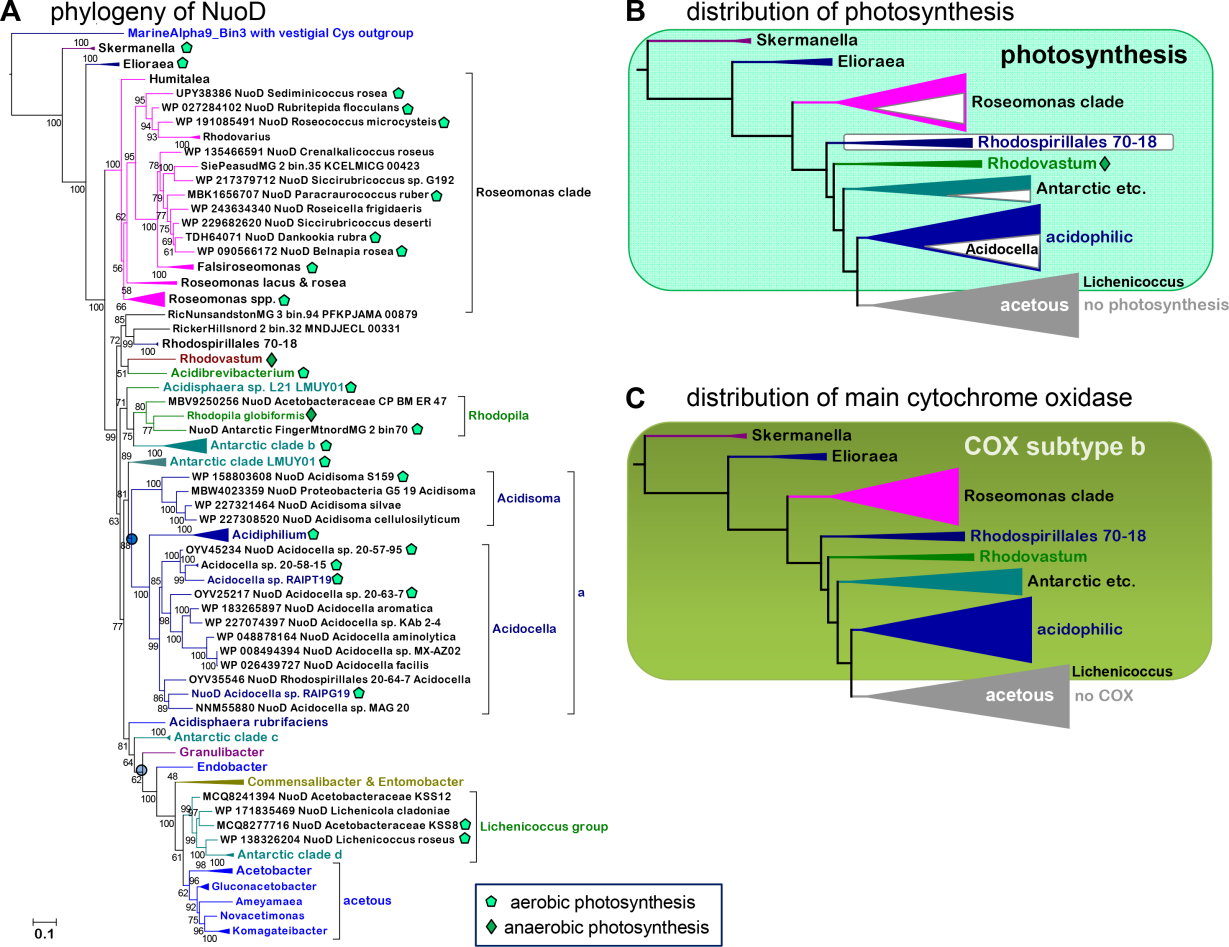
(A) Phylogenetic tree of *Acetobacteraceae* NuoD. The ML tree was reconstructed using the NuoD subunit of Complex I, which has a strong phylogenetic signal (34). NuoD trees can be rooted in homologs that preserve vestigial ligands such as those of MarineAlpha9 MAGs (34) used here as the outgroup. The alignment was extended to representative NuoD homologs from Antarctic MAGs (21) and MAGs from Patagonia (Table 2; Table S1), as shown in Fig. 2A; it had 110 sequences with 429 amino acid sites, 28.4% of which were constant. The tree was reconstructed with the EX_EHO mixture model and was representative of various ML trees obtained with different taxonomic samplings and substitution models. Only the species or strain name is indicated for each protein.orThe letter a indicates the acidophilic clade with *Acidocella* as in Fig. 1A. The blue round symbols indicate the nodes for the sister clades of acidophilic and acetous plus their subtending taxa. Photosynthesis, indicated by the symbols shown in the legend at the bottom, was deduced from the presence or absence of PufML genes as well as those of Form I Rubisco (43). (**B)** The distribution of photosynthetic traits along the phylogeny of *Acetobacteraceae* was sketched over a compressed version of the ML tree in panel A. The distribution was assessed by the presence/absence analysis of key photosynthetic traits (Table S2; Fig. S4). White triangles within clades represent taxa without photosynthetic traits like Rhodospirillales 70–18. The green rhomboid symbol indicates anaerobic photosynthesis as in panel A. (**C)** The distribution of the major cytochrome oxidase, A1 type COX operon subtype b (20), was sketched over a compressed version of the tree in panel A.

The second largest group of *Acetobacteraceae* revolves around *Roseomonas*, for which there is an increasing number of taxa and closely related genera (1) that seldom have an acidophilic character, or mildly so (Table 1). *Roseomonas* and related taxa regularly form a compact clade that branches immediately after the clade of *Elioraea* (Fig. 1A, 2A, and 3A), which we confirmed to be the basal genus of *Acetobacteraceae* in phylogenetic trees reconstructed with concatenated ribosomal proteins [Fig. 1A, cf. references (3 – 5, 34)] and single marker proteins (Fig. 2A and 3A). Our results do not lend support to the proposal of a separate family for *Elioraea* (42). In expanded trees reconstructed with 16S rRNA sequences, some members of the *Roseomonas* clade, such as *Roseococcus,* form separate branches [Fig. S1A, cf. reference (41)]. This situation is most likely derived from the limited resolution of the very conserved sequences of rRNA. Indeed, phylogenetic trees reconstructed with single marker proteins (Fig. 2A and 3A, as well as Fig. S1C) or concatenated ribosomal proteins (Fig. 1A; Fig. S1D) invariably show a compact *Roseomonas* clade.

The central part of phylogenetic trees of *Acetobacteraceae* includes non-acetous acidophilic taxa of the family. Some of such taxa often cluster in a sister clade to the acetous taxa, while *Acidocella* and *Acidiphilium* form a basal branch that is closer to the *Roseomonas* clade than to the acetous clade [Fig. 1A, cf. references (1 – 5, 36)]. However, this common branching pattern depends upon the phylogenetic marker and taxonomic sampling used to reconstruct the trees. Even minimal differences in the taxonomic sampling of concatenated ribosomal proteins seem to alter the relative position of the acidophilic clade, as summarized in Fig. 1B. This was not the case for phylogenetic trees reconstructed with equivalent or larger taxonomic samplings of the AtpD or NuoD protein (Fig. 2A and 3A) and also with 16S rRNA sequences [Fig. S1A, cf. reference (41)]. These trees consistently showed the acetous clade in a sister position to the clade of acidophilic taxa, including *Acidocella*, *Acidiphilium,* and also *Acidisoma*, while *Rhodovastum* and other predominantly photosynthetic taxa generally formed a basal clade (Fig. 2A and 3A). Trees reconstructed with cytochrome *b* sequences clearly confirmed the sisterhood of the acidophilic and acetous clades (Fig. S1C), which would be consistent with the shared bioenergetic trait of the non-functional *bc*_1_ complex in these sister clades (Fig. 2C). Presumably, *bc*_1_ complex genes are retained in *Acetobacteraceae* predominantly using ubiquinol oxidases (9, 12 – 14) because the proteins coded by such genes fulfill a structural role in stabilizing Complex I (44), which is fundamental for their physiology (13 – 15). We subsequently used the phylogenetic tree of the NuoD subunit of Complex I as a framework to evaluate the evolutionary steps determining the respiratory chain changes in various *Acetobacteraceae* (Fig. 3A).

To sum up, two firm considerations emerged from our comprehensive analysis of the *Acetobacteraceae* phylogeny. The first is that the *Roseomonas* clade likely constitutes a separate subdivision of *Acetobacteraceae*, which we propose to designate the family *Roseomonadaceae*. The second is that *Acidisoma* taxa cluster with *Acidocella* and *Acidiphillium* taxa, together forming a newly defined clade of acidophilic members of *Acetobacteraceae*. We will preliminarily call this clade “acidophilic” to distinguish them from the generally less acidophilic but predominantly photosynthetic taxa such as *Rhodovastum*.

### Photosynthetic traits are widespread among *Acetobacteraceae*

We have already mentioned that diverse members of *Acetobacteraceae* possess photosynthetic traits, for example, *Rhodovastum*. However, such traits have been scantly evaluated in the literature. For instance, a recent survey of *Roseomonas* taxa (1) did not consider photosynthetic traits, which we estimate to be present in about 40% of current taxa forming the *Roseomonas* clade (cf. Table 1). Here, we evaluated in granular detail the distribution of photosynthetic traits among *Acetobacteraceae*. Photosynthesis has been previously associated with *Acidiphilium* (45), *Rhodopila*, *Rhodovastum* (22, 46), and *Acidisphaera* (40, 47 – 49). However, our analysis indicates that only one-half of the genomes currently listed under *Acidisphaera* (six in GTDB: https://gtdb.ecogenomic.org/searches?s=al&q=g_Acidisphaera, accessed on 22 May 2023) possess photosynthetic traits. Indeed, the genus *Acidisphaera* is polyphyletic: non-photosynthetic *Acidisphaera* sp. S103 clusters with *Rhodopila* (Fig. 1A and 2A), while photosynthetic *Acidisphaera* sp. L21 (Table S2) clusters with various Antarctic MAGs (Fig. 1A, 2A, and 3A). The latter finding is consistent with the separate classification of this taxon under the LMUY01 genus in GTDB taxonomy (28) and illustrates the widespread distribution of photosynthetic traits among *Acetobacteraceae* MAGs from Antarctic environments (Table 2). Originally, the majority of Antarctic MAGs were reported to cluster with Rhodospirillales sp. 70–18 (21), a non-photosynthetic genus previously considered to be part of Acetobacterales (28, 50). Our analysis indicated that only a couple of Antarctic MAGs actually cluster with Rhodospirillales sp. 70–18 (Fig. 3A, cf. Table 2), while about one-half of the other MAGs cluster with established photosynthetic *Acetobacteraceae* such as *Rhodopila* (clades a, b, and LMUY01, Table 2). Conversely, two non-photosynthetic Antarctic MAGs (clade d, Table 2) cluster within the group of *Lichenicoccus* (36), which includes the recently reported (51) *Acetobacteraceae* KSS8 and KSS12 (Fig. 1A and 2A). Antarctic MAGs did not cluster within the acidophilic clade, which we expanded by adding novel taxa with photosynthetic traits: Proteobacteria bacterium isolate G5_19, re-classified in the *Acidisoma* genus, three *Acidocella* from acidic environments of Patagonia possessing photosynthetic traits, and various Rhodospirillales MAGs that we re-classified as members of either the *Acidiphilium* genus or the *Acidocella* genus (Table S1). Overall, our increased taxonomic sampling indicates that photosynthetic traits pervade the phylogenetic space encompassing *Rhodovastum* to *Lichenicoccus*, despite multiple instances of gene loss in closely related taxa. Moreover, the earliest branches of the *Acetobacteraceae* phylogeny, *Elioraea* and *Roseomonadaceae*, have a pervasive presence of photosynthetic traits too (Table 1; Fig. 3B). Therefore, we surmise that the common ancestor of *Acetobacteraceae* probably possessed photosynthetic traits. This is a novel concept in the phylogeny of *Acetobacteraceae* [cf. references (2) and (4, 36)], which deserved further detailed analysis.

**Fig 4:**
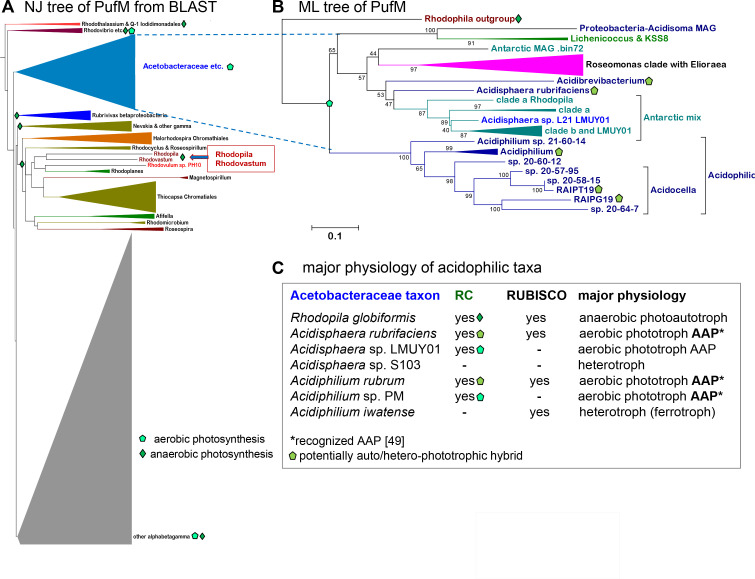
Phylogenetic trees of photosynthetic marker proteins. (A) The phylogenetic NJ tree of PufM, the large subunit of the photosynthetic reaction center (RC), was rendered with the program MEGA5 (34). The tree was obtained directly from PSI-BLAST1000 of WP_138324303 PufM of *Lichenicoccus* against the whole nr database (accessed on 13 September 2022). Over 96% of the hits included proteins from alpha-, beta-, and gammaproteobacteria; the few proteins from other classes were removed for building the tree. The outgroup formed by *Caenispirillum salinarum* PufM was cut off from the graphical presentation (see Fig. S3A for an expansion of the top part of this tree containing also the outgroup). The clade comprising all the anoxygenic aerobic phototrophs of *Acetobacteraceae* is early branching, rather distant from the clade comprising *Rhodovastum* and *Rhodopila*, which cluster with photosynthetic *Magnetospirillum* and *Rhodoplanes* spp. that also have the rare trait of rhodoquinone (22, 52). These proteins are embedded in a large clade dominated by photosynthetic gammaproteobacteria of the Chromatiales order, in partial agreement with previous phylogenies (40). The large gray clade containing later diverging PufM proteins is cut off at its middle. (**B)** The ML tree was constructed using an alignment of 50 PufM sequences that combined most representatives of the clade of photosynthetic *Acetobacteraceae* in panel A with non-redundant homologs of environmental MAGs from Antarctica (21) and Patagonia (this work). The alignment had 344 amino acid sites (34% of which were constant), and the tree was reconstructed with the best-fit model (39) LG. Very similar results were obtained with the mixture model EX_EHO. *Rhodopila* was used as the outgroup, given its different photosynthetic character shared with *Rhodovastum* (panel A). (**C)** Combinations of photosynthetic and carbon fixation traits define the major physiology of acidophilic *Acetobacteraceae*. The table presents an extract of the data shown in Fig. S4 and Table S2. The type of major physiology was taken from microbiology and biochemical data (45, 49, 52) or deduced from the combined traits of photosynthesis (RC) and Form I Rubisco [cf. references (26, 43)].

We undertook the phylogenetic analysis of multiple proteins defining photosynthetic traits, starting with the largest subunit of the photosynthetic reaction center (RC), PufM (Fig. 4A and B; Fig. S3A). BLAST searches against the whole nr database have shown that PufM proteins of most *Acetobacteraceae* cluster in a clade that appears to be early branching (Fig. 4A). However, the PufM proteins of *Rhodovastum* and *Rhodopila* cluster in a separate branch containing various purple bacteria of the alphaproteobactera class intermixed with gammaprotobacteria such as *Halorhodospira* (Fig. 4A). All these bacteria have the physiology of anaerobic photosynthesis; namely, they use phototrophic autotrophy when light is available and oxygen is scarce, but revert to heterotrophic aerobic metabolism in the dark (40). Among *Acetobacteraceae*, only *Rhodovastum* (22) and *Rhodopila* (47, 52) have the same physiology, while *Eliorea thermophila* has been reported to grow phototrophically using substrates that typically sustain autotrophic growth with anaerobic photosynthesis (42, 53). This versatile phototrophic phenotype has been linked to the presence of Form I ribulose 1,5-bisphosphate carboxylase-oxygenase (Rubisco), an enzyme usually associated with photo-autotrophy (40, 43, 49) that is present only in *E. thermophila* among *Elioraea* species (53). Indeed, Rubisco has been used as a proxy to differentiate photo-autotrophic from photo-heterotrophic (40) or aerobic anoxygenic phototrophs (AAPs) (26, 43). AAP cannot grow phototrophically under anaerobic conditions because they lack Rubisco (43).

Here, we used the absence of Rubisco to define *bona fide* AAPs as in previous works (40, 43) but realized that this criterion would exclude bacteria previously recognized as AAPs, even if their genomes possess both subunits of Form I Rubisco, for example, *Acidisphaera* (43) (Fig. 4C). Remarkably, all possible combinations of photosynthetic traits with Rubisco are present in related acidophilic *Acetobacteraceae* (Fig. 4C; Fig. S4). This is consistent with the emerging picture of Rhodospirillales AAPs that have Rubisco and the whole Calvin cycle for carbon fixation (54). There also are taxa without photosynthetic traits that contain a catalytically active Form I Rubisco, as in the case of *Acidiphilium iwatense* (Fig. 4C; Fig. S4). Rubisco and its associated Calvin cycle can sustain other forms of autotrophic physiology, for example, methylotrophy (55). This explains the presence of two closely related Rubisco isoforms in *Acidimonas methanolica* (Fig. S4), a member of the acetous clade that uniquely has methylotrophic physiology (55). By analogy, it is possible that *Acidiphilium iwatense* and other *Acetobacteraceae* retain Rubisco to enable growth with chemolithotrophic pathways, in particular iron oxidation (ferrotrophy), which is widespread among acidophilic *Acetobacteraceae* (Table S2). Notably, *Acidiphilium iwatense* is one of the few acidophilic taxa that does not have a complete operon for the most common type of COX in alphaproteobacteria (20) (Fig. S4). This situation reflects a peculiar correlation between photosynthetic traits and COX (Fig. 3), which will be discussed next.

### Photosynthetic traits correlate with the distribution of cytochrome oxidase

Early in our study, we realized that the distribution of photosynthetic traits among *Acetobacteraceae* essentially matches that of cytochrome oxidase (Fig. 3 and 4; Fig. S2 to S5), specifically of A1-type COX operon subtype b (20). Genomes that do not contain this operon have sometimes A2-type COX operon subtype a-I (Fig. S2B; Table S2), which is normally associated with nitrogen metabolism (20). Intriguingly, the distribution of COX operon subtype b stops at the phylogenetic junction of the acetous clade (Fig. 3C; Fig. S2B and C). However, there are acetous taxa that retain genes for the metabolism of photosynthetic carotenoids (Fig. S4), which is generally associated with photo-autotrophy (40). Indeed, the various genes responsible for the biosynthesis of carotenoids form part of the photosynthetic gene cluster, including the RC subunits and various enzymes for the biosynthesis of porphyrins and chlorophyll (26). The overall panorama of trait-defining proteins associated with photosynthesis in *Acetobacteraceae* (Fig. S4) suggests a pattern of differential loss with retention of vestigial characters, as in the case of Rubisco (Fig. 4C). This pattern is coupled to the loss of COX in several phylogenetically separate genera (Fig. 3; Fig. S4), a situation that differs from that present in Rhodobacterales (26). In the latter lineage, the loss of photosynthesis is rarely associated with that of COX but is generally linked to that of other proteins of the photosynthetic gene cluster, including those involved in carotenoid biosynthesis (26). The simplest explanation for such differences is that the distribution of photosynthetic and COX genes does not derive from the same evolutionary pattern in *Acetobacteraceae* and Rhodobacterales.

Lateral gene transfer (LGT) has been shown to be the dominant factor in the distribution of photosynthetic traits among Rhodobacterales (26, 40). Indeed, the photosynthetic gene cluster is located in plasmids of several Rhodobacterales (26) but not in *Acetobacteraceae* (45). The most evident LGT cases of photosynthetic traits in *Acetobacteraceae* regard the Rubisco present in *Elioraea thermophila* and two environmental MAGs, which belong to Form IA typical of gamma- and betaproteobacteria (27). In the case of PufM, there are likely LGT instances from *Acetobacteraceae* to other taxa, either somehow related, such as *Skermanella*, or clearly unrelated, such as deltaproteobacteria MAGs (Fig. S3A). This situation and the early branching position of the clade including most *Acetobacteraceae* PufM (Fig. 4A) suggest that photosynthesis entered early in the ancestral lineage of the family, perhaps concomitantly with the adaptation to stable levels of oxygen in the environment. The latter possibility emerged from combining our observations with established evidence regarding the evolution of photosynthesis in Proteobacteria (26, 40, 43, 49). The first piece of evidence is that anaerobic photo-autotrophy is ancestral to aerobic phototrophy (40); namely, photo-autotroph *Rhodovastum* is early divergent vs AAP such as *Acidiphilium*. Phylogenetic trees of the whole family sustain this (Fig. 2A and 3A, as well as Fig. S1A; Fig. S3B and C). The second piece of evidence is that RCs containing the tetraheme cytochrome *c* subunit, PufC, are more ancient than those having the PufX subunit instead (26, 40, 56). Connected to the latter, anaerobic photo-autototrophs have PufC proteins displaying a conserved Cys toward the N-terminus, which is used for post-translational attachment of a lipid anchor. Conversely, AAP taxa generally have PufC proteins without such a residue and remain attached to the membrane via an N-terminal transmembrane helix (56). This situation is present in *Rhodopila* (56) and the great majority of *Acetobacteraceae*, with the exception of a few members of the *Roseomonas* clade (Fig. S3B). Finally, the presence of Form I Rubisco in taxa that have been phenotypically characterized as typical AAPs such as *Acidisphaera* (43, 47, 49, 52) suggests that *Acetobacteraceae* evolved around the time of the transition from anaerobic to aerobic phototrophy, a transition that left extant taxa with a hybrid physiology (Fig. 4B). Clearly, this must have occurred as an adaptation to increasing and stable levels of oxygen in the environment (43).

COX evolution equally reflects bacterial adaptation to increasing levels of oxygen in the environment (18 – 20). Moreover, COX activity is required to maintain RC components that are sufficiently oxidized during photosynthetic electron transport in AAPs (43, 57, 58). This requirement may rationalize the correlation in the distribution of COX and photosynthetic traits (Fig. 3B and C; Table S4). The molecular signatures determining the different redox properties of RC components in AAPs remain elusive (43). However, the recently reported structure of the *Rhodopila* RC (56) may provide new clues. In particular, the C-terminal part of PufM extends at the periplasmic side of the RC complex, covering the PufC part that binds heme 3 (Fig. S5A). This cytochrome *c* heme directly transfers electrons to the photo-oxidized special pair of bacteriochlorophylls (56). The C-terminal part of PufM is missing in photo-autothrops such as *R. sphaeroides* and *R. rubrum*, the RC of which lacks the PufC subunit (Fig. S5B). Conversely, the PufM sequences of photosynthetic *Acetobacteraceae* and many other AAPs have a C-terminus with a length equivalent to that of *Rhodopila*. These sequences generally maintain the chemical character of PufC-interacting residues and may thus influence the electron transport properties between PufC and the RC (Fig. S5).

In sum, traces of a photosynthetic past are present in genomes of extant non-photosynthetic *Acetobacteraceae*, reflecting a pattern of vertical inheritance that is peppered with differential loss and transition states in overall physiology.

### Loss of photosynthesis may be compensated by additional enzymes reducing ubiquinone

**Fig 5:**
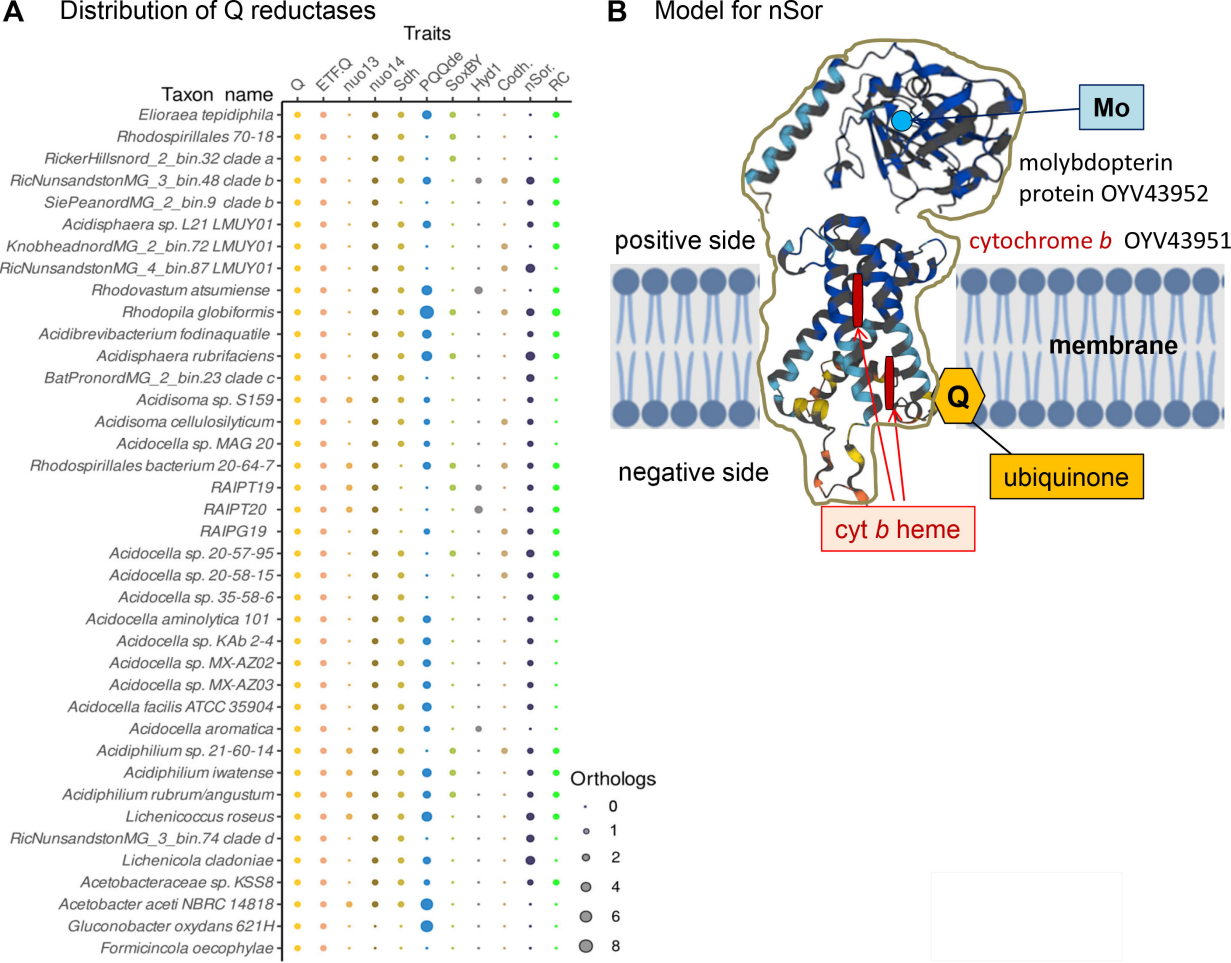
(A) The distribution of traits for ubiquinone (Q) reduction among representative members of the *Acetobacteraceae* family was rendered in dot plot format (19). The presence of multiple orthologs for the proteins defining each trait is shown by dots of different sizes as indicated in the legend at the bottom. The nuo13 and nuo14 operons of Complex I were in separate columns as in Table S2. The two forms of aerobic carbon monoxide dehydrogenase (Codh#) were identified from sequence signatures and genomic clusters (63). (B) Model for the transmembrane di-heme cytochrome *b* OYV43951 related to *E. coli* YdhU that could reduce Q in the novel Sulfoxide-Q oxidoreductase (nSor in panel A)—with AlphaFold (64, 65) and the addition of hemes, Q and the membrane. This enzyme is the sole member of the sulfite oxidase sub-family (61) with associated membrane cytochrome *b* in *Acidocella* (see text).

If the ancestor of *Acetobacteraceae* was originally photosynthetic, it follows that the loss of the RC function of ubiquinone (Q, Fig. 2B) reduction would be compensated by other Q reductases to maintain a redox balance in the respiratory chain. To verify such a possibility, we investigated the presence of a variety of Q reductases in the genomes of *Acidocella* and other acidophilic taxa (Fig. 5A). The various dehydrogenases that are constitutively present in basically all genomes are electron transfer flavoprotein dehydrogenase (ETF-Q) and NADH-quinone reductase (Complex I, Fig. 5A), in agreement with previous surveys (14, 59, 60). The SoxBY markers, representing alternative Q reduction pathways associated with sulfur oxidation (45), displayed instead a patchy distribution, not dissimilar to that of type 1 hydrogenase and carbon monoxide dehydrogenase (Codh) (Fig. 5A). Indeed, aerobic photosynthesis helps a variety of bacteria to survive in deserts and other extreme, oligotrophic environments (54, 61). Ultimately, the distribution pattern and the number of pyrroloquinoline (PQQ)-dependent Q reductases emerged as one of the most widespread traits compensating for the loss of RCs (Fig. 5A). PQQ-dependent dehydrogenases are the dominant metabolic enzymes of acetous taxa (2, 4, 14, 15, 59, 60); except for *Rhodopila* (52), these enzymes have a limited presence in other *Acetobacteraceae* (Fig. 5A), despite the widespread distribution of the operons for the biosynthesis of the PQQ cofactor (62). Intriguingly, *Acidocella* taxa that do not have photosynthetic traits possess two or more PQQ-dependent Q reductases (Fig. 5A), sustaining the possibility that these enzymes may compensate for the loss of Q reduction by RC.

During our search for additional Q reductases, we discovered a putative redox enzyme that is widespread among *Acidocella* taxa. We named this enzyme nSor, standing for novel Sulfoxide-Q oxidoreductase (Fig. 5B). It contains two different proteins coded by adjacent genes resembling the *yedYZ* of *Escherichia coli*, recently re-named MsrPQ for it constitutes a bacterial methionine sulfoxide reductase (66, 67). This enzyme belongs to the group of sulfite oxidases (68) and helps in quenching oxidative damage to proteins by reducing methionine sulfoxides (66, 67). Although MsrPQ normally oxidizes ubiquinol to reduce methionine sulfoxides in the periplasm (68), nSor likely functions as a Q reductase because its membrane di-heme cytochrome *b* is structurally similar (Fig. 5B) to the Q-reducing cytochrome *b* subunit of formate dehydrogenase (69). This hypothesis requires further experimental tests.

### Phyletic patterns reveal diversification and duplication of *Acetobacteraceae bo*_3_ ubiquinol oxidases

**Fig 6:**
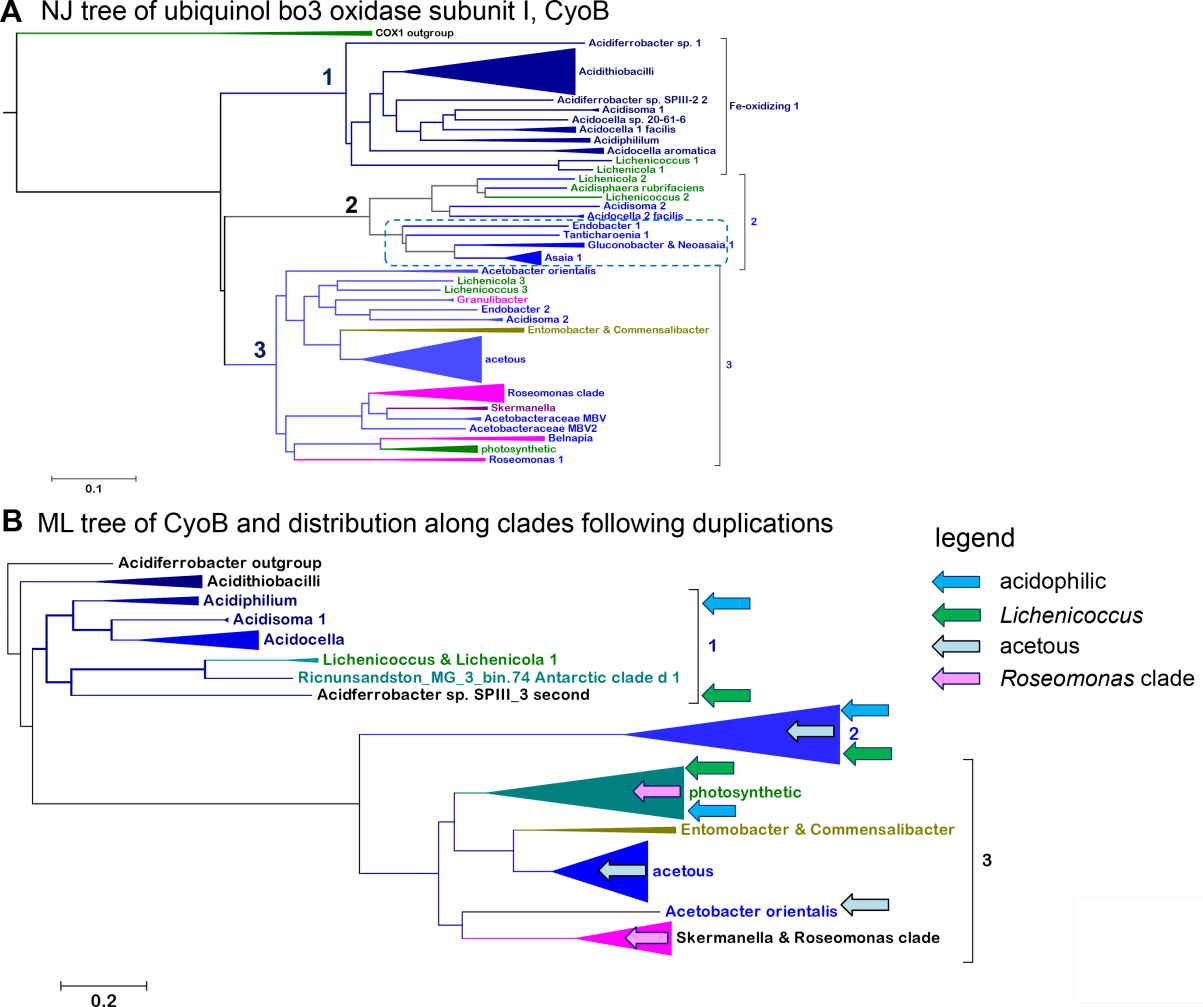
Phylogeny and distribution of cytochrome *bo*_3_ ubiquinol oxidases. (**A)** NJ tree of 145 representative CyoB proteins retrieved from a wide BLAST search of *Asaia* CyoB1 (59) WP_062164020 against all alphaproteobacteria. Three paralog COX1 proteins were used as the outgroup. The dashed box indicates the subclade of acetous taxa with a second CyoB included in clade 3. (**B)** Compressed view of an ML tree reconstructed with 128 CyoB sequences containing 730 amino acid sites and the EX_EHO model. The arrows indicate the duplication of proteins from major clades. The arrow of *Lichenicoccus* includes also *Lichenicola*. Figure S7 shows an expanded similar tree with annotated support values.

We next assessed the phyletic patterns and phylogeny of bioenergetic traits for the oxidation of ubiquinol. Soluble cytochrome *c*_2_ is reduced by the *bc*_1_ complex, which is the major partner of RCs in re-oxidizing ubiquinol [Fig. 2B, cf. references (26, 40, 49)]. However, *Acetobacteraceae* genomes contain other enzymes for ubiquinol oxidation that utilize oxygen as an electron acceptor, notably the *bo*_3_ ubiquinol oxidase (13, 14, 45, 59, 60) (Table S2). The most recent study on the origin of *bo*_3_ ubiquinol oxidase among *Acetobacteraceae* concluded that the *bo*_3_ oxidase of *Acidiphilium* was acquired from *Acidithiobacillus* spp. sharing the same extremely acidic habitats (45). We agree with this conclusion since our phylogenetic trees of the catalytic subunits CyoB (Fig. 6) and CyoA (Fig. S6) invariably show a close vicinity of *Acidiphilium bo*_3_ oxidase with homologs from *Acidithiobacillus* and *Acidiferrobacter* spp.

The clade of *Acidiphilium* CyoB, named clade 1 here, also includes proteins from *Acidocella*, *Acidisoma*, *Lichenicoccus,* and *Lichenicola* (Fig. 6; Fig. S7). Remarkably, the genomes of *Lichenicoccus* and *Lichenicola* contain two additional CyoB proteins belonging to separate operons segregating in different clades, labeled 2 and 3 here (Fig. 6). Previously, it was reported that multiple operons for the *bo*_3_ ubiquinol oxidase were specifically present in the *Asaia* genus among *Acetobacteraceae* (59). However, we found that some *Acidocella*, including our newly reported MX-AZ03 (Fig. S7), one *Acidisoma*, *Acidisphaera rubrifaciens,* and four acetous genera also have a second operon clustering in clade 2 with CyoB-2 from *Asaia* (59) (Fig. 6; Fig. S7). Clade 2 additionally includes the *bo*_3_ oxidase from some Antarctic MAGs and is separate from clade 3, which encompasses the second *bo*_3_ oxidase of acetous taxa, as well as the third *bo*_3_ operon of *Lichenicoccus* and *Lichenicola* (Fig. 6B; Figs. S6 and S7). Clade 3 is part of a very large cluster of *bo*_3_ oxidases from diverse proteobacteria (Fig. S6A), consistent with earlier studies (9, 13, 59). Distance trees obtained from BLAST searches of the nr database—accessed on 27 October 2022—are basically consistent with this picture but clearly show the early branching position of clade 1 proteins from acidophilic taxa and the *Lichenicoccus* group clustering together with those of acidithiobacilli and *Acidiferrobacter* spp. (Fig. S6A). This finding suggests that *Acetobacteraceae* initially acquired their *bo*_3_ oxidases from either *Acidithiobacillus* or *Acidiferrobacter* species and subsequently transmitted them to other lineages. The process was associated with operon duplication in the genome of the ancestor of some *Acidocella* strains, leading to the separation in different *bo*_3_ oxidases, one of which clusters in clade 2 (Fig. 6; Fig. S7). Instead, the second isoform clusters within a very large clade comprising the *bo*_3_ oxidases of other proteobacteria (Fig. S6A). Our results additionally suggest a late acquisition of *bo*_3_ oxidase by early branching *Acetobacteraceae* such as *Rhodopila* (Fig. 6). The question that then emerges is this: what has driven the diversification of *bo*_3_ oxidase from the original operon acquired from iron oxidizers?

### Derangement of the *bc*_1_ complex fueled *bo*_3_ oxidase diversification among *Acetobacteraceae*

**Fig 7:**
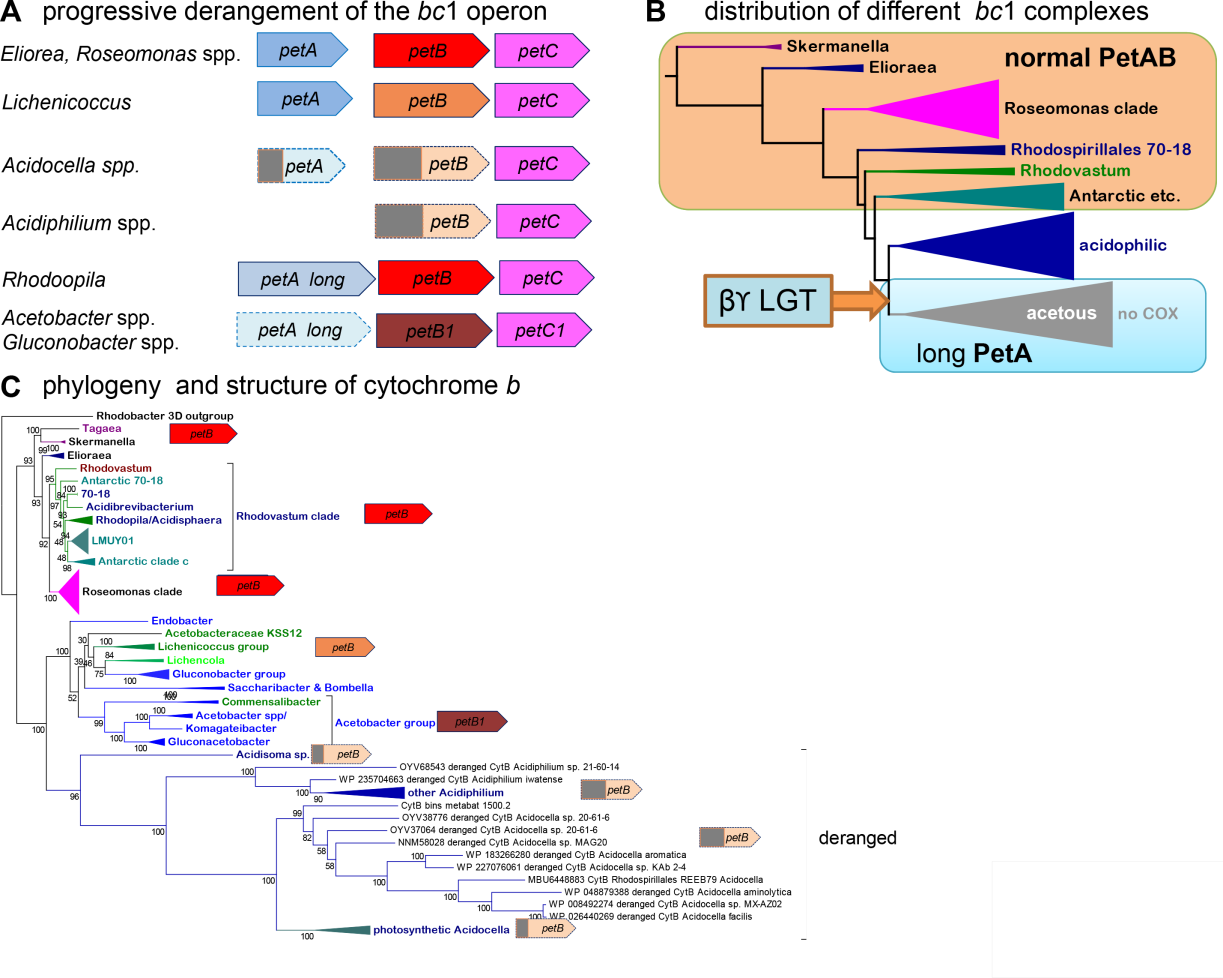
Derangement of the *bc*_1_ complex operon and proteins. (A) Schematic representation of the *petABC* operon. The lack of conserved ligands for metal centers in the proteins is represented by gray squares. The pale bluish color of the long ISP of acetous taxa indicates the lower redox potential that these proteins likely have (see text and Fig. S9). The pale blue symbol with a dashed contour indicates that the *petA* gene is separate from the rest of the operon. Fully functional cytochrome *b* is indicated by the bright red *petB* symbols, while the cytochrome *b* of acetous taxa is indicated by the dark red *petB* symbol. The cytochrome *b* of members of the *Lichenicoccus* group is indicated by an orangey *petB* symbol because of the presence of unusual amino acid substitutions in key regions of the proteins. (B) Distribution of the various forms of key proteins of the *bc*_1_ complex: PetA, Rieske ISP, and PetB, cytochrome *b*. The background phylogenetic tree of *Acetobacteraceae* was taken from Fig. 3A. The large arrow indicates the likely origin of the long form of ISP present in acetous taxa, which seems to be part of a massive LGT wave from gamma- and related betaproteobacteria, as discussed earlier (34, 70, 72). (C) The phylogenetic ML tree of cytochrome *b* was obtained with the best-fit LG model and gamma = 4 (39) using an alignment of 128 sequences expanded from that used for the tree in Fig. S1C. The different forms of the cytochrome *b* protein with partial or complete loss of the His ligands of cyt *b*_H_ (Fig. S1B) are indicated by symbols as in panel A.

To find the answer to the above question, we explored phyletic and molecular aspects of the *bc*_1_ complex (37, 70). Our results indicated a functional degeneration of the *bc*_1_ complex in various *Acetobacteraceae*, as shown in Fig. 7 (see also Fig. S1B and S8). It has been reported previously that the genome of *Acetobacter* spp. contains the gene for a deranged COX1-like protein lacking all the conserved residues binding the metal cofactors (13). However, this gene has been retained as part of a cluster containing the gene for CtaB, the enzyme that produces cytochrome *o* for the *bo*_3_ ubiquinol oxidase (13), probably fulfilling a structural role in stabilizing CtaB. Therefore, *Acetobacteraceae* genomes maintain genes for respiratory proteins that are deranged, a situation that is particularly relevant for the *bc*_1_ complex, which may be required for the stability of Complex I (44). This enzyme is of questionable function in acetous taxa (13, 15, 60) and even more in *Acidiphilium* (45), which lacks the gene for ISP (Fig. S8) and also the two conserved histidines that bind the cytochrome *b*_H_ [Fig. S1B, cf. reference (70)]. We found a similar situation in *Acidocella* genomes, which contain an ISP subunit that is even more deranged than cytochrome *b* (Fig. 7; Fig. S8). *Acidocella* ISP lacks three or four ligands of the Fe_2_S_2_ cluster but maintains the couple of cysteine residues that hold the cluster together by a disulfide bond (71) (Fig. S8C).

Conversely, the long ISP typical of acetous taxa and gammaproteobacteria (71) was found also in *Rhodopila*, Antarctic MAGs of clade c, and the *Lichenicoccus* group (Fig. S9). This long version of ISP generally shows the substitution of Tyr165 with Phe (Fig. S8C), which prevents the H-bond that Tyr165 normally entertains with a cluster ligand (71), thereby significantly reducing the activity of the *bc*_1_ complex (73, 74). It is therefore likely that the *bc*_1_ complex encoded by the split *petA* and *petBC* operons in *Lichenicoccus*, *Lichenicola*, *Acetobacter,* and related acetous taxa (13 – 15, 36, 72) has reduced catalytic activity. Intriguingly, the genome of the single *Acidisoma* taxon that retains the *petABC* operon shows intermediate features, consistent with the intermediate position of this taxon in the phylogenetic trees of the ISP protein (Fig. S9) and cytochrome *b* (Fig. 7C; Fig. S1B), as well as in those of cytochrome *c*_1_ (Fig. S10) and Cox15 (Fig. S11). In all such trees, the sisterhood of the acidophilic and acetous clades is strongly supported, as shown in Fig. 3A; Fig. S1C.

We undertook phylogenetic analyses of Cox15 (35) because its gene is present in the genome of *Acetobacter* spp. (13) and is necessary for A2-type COX subtype a-I present in some acetous taxa (Fig. S2; Table S2). Many other acetous taxa possess a cytochrome *c* peroxidase that may undertake the re-oxidation of reduced cytochrome *c*, as proposed in *Acetobacter aceti* (14) (Fig. S2A). Such a possibility is probably not as efficient as COX; therefore, the respiratory chain of *Acetobacter*, *Gluconobacter,* and related acetous taxa may be physiologically deranged at the level of cytochrome *c* (13). This indicates that both acetous and acidophilic taxa share a functionally deranged respiratory chain, as discussed earlier. Ubiquinol oxidation might be compensated by the cytochrome *bd* ubiquinol oxidases that are widespread among *Acetobacteraceae* (9, 13, 14) (Fig. S4; Table S2). However, the puzzling question remains: why do most *Acidocella* and *Acidiphilium* taxa possess an apparently functional cytochrome oxidase while having a deranged *bc*_1_ complex? Besides the mentioned structural role in stabilizing Complex I (44), a possible answer resides in iron oxidation physiology (Fig. S4).

### Pathways for iron oxidoreduction bypass deranged *bc*_1_ complexes in reducing *c*-cytochromes

**Fig 8:**
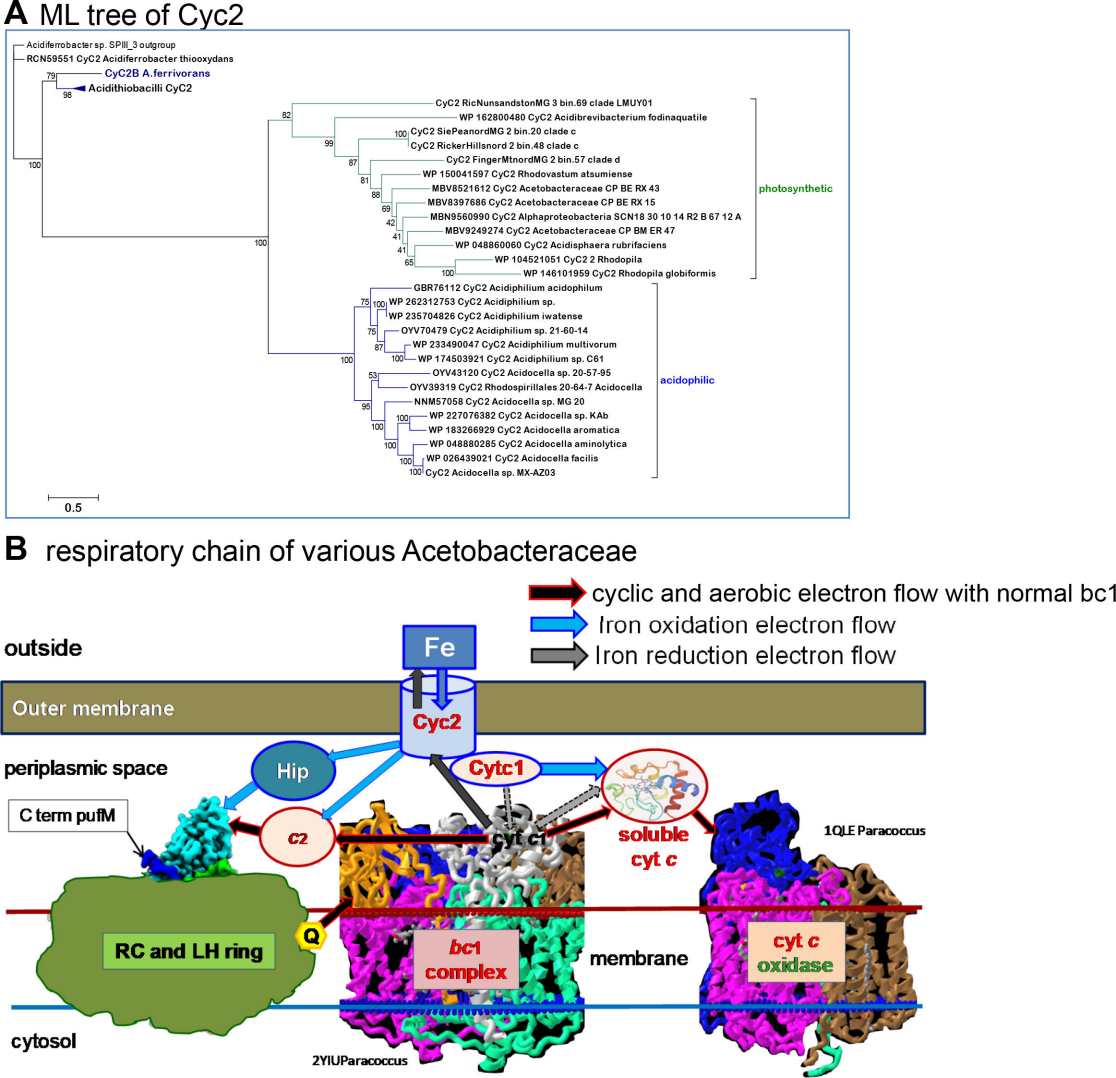
Distribution of ferrotrophy and its role in the respiratory chain of *Acetobacteraceae*. (A) Phylogeny and distribution of outer membrane Cyc2 among *Acetobacteraceae*. The ML tree was obtained from an alignment of 34 homologs of Cyc2B ACK78881 of *Acidithiobacillus ferrooxidans* involved in the reduction of extracellular ferric iron (19, 77), which were retrieved from a PSI-BLAST search against some acidithiobacilli, *Acidiferrobacter* spp., and all *Acetobacteraceae*. The alignment was manually refined and contained 574 amino acid sites, 15% of which were constant and included the CxxCH motif near the N-terminus for heme binding (78). The tree was reconstructed with the best-fit model (39) LG and gamma = 4. (B) Detailed illustration of the central part of the respiratory chain of acidophilic *Acetobacteraceae*. The illustration presents the possible connection of the oxidoreduction of extracellular iron (Fe) to the electron wire pivoting on Cyc2 (75). The different pathways of electron flow are indicated by the differently colored arrows. The 3D structure of the RC surrounded by the light-harvesting (LH) annulus of *Rhodopila* was modified from a figure in reference (56), while those of the dimeric form of the *Paracoccus bc*_1_ complex (81) and of *Paracoccus* COX (82) have been rendered with iCn3D Structure Viewer. Q represents the reduced form of Q, ubiquinol. The C-terminal part of the PufM protein sticking out in the periplasmic space close to the PufC protein (Fig. S5) is shown on the left.

*Acidiphilium* species have been known for a long time to reduce ferric iron under micro-aerobic conditions (75), while *Acidocella aromatica* can grow using the same pathway under anaerobic conditions (76). In acidithiobacilli, the pathway of ferric iron reduction consists of isoforms of the outer membrane Cyc2 and periplasmic Cyc1 *c*-type cytochromes, which are directly involved in the opposite reaction, the oxidation of ferrous iron (19, 75, 77). Cyc2 proteins have been shown to resemble a beta-barrel porin with the cytochrome *c* heme exposed to the periplasmic space; they are more widely distributed than previously thought (78). We have confirmed the presence of Cyc2 homologs also in *Acidocella* MX-AZ03 and one of the *Acidocella* MAGs reported here, as well as in early branching *Acetobacteraceae* such as *Acidibrevibacterium* (Fig. 8A) and some Antarctic MAGs (Fig. S4). Notably, the gene for Cyc2 is often adjacent to another encoding either a mono- or di-heme *c*-type cytochrome (Fig. S4), which likely represents a functional homolog of the Cyc1 protein involved in iron oxidation (75, 77, 78). In acidithiobacilli, HiPIP is another component of the electron wiring connecting the oxidation of extracellular ferrous iron with *c*-cytochromes (19, 75, 78), although its role remains unresolved. Conversely, HiPIP proteins assist cyclic electron flow in photosynthetic purple bacteria (79), as well as in photoferrotrophy (80). Our analysis indicates that HiPIP must be crucial for sustaining aerobic photosynthesis in *Acidisphaera* sp. L21, which lacks genes for cytochrome *c*_2_ and its homologs (Fig. S4). HiPIP can also function as the oxidizing substrate for the *bc*_1_ complex (79), but it is present in acidophilic taxa that have deranged forms of the complex (Fig. S4).

Notably, the *bc*_1_ complex can transfer electrons from ubiquinol to cytochrome *c*_1_ even when cytochrome *b*_H_ is blocked by inhibitors such as antimycin (83). The absence of cytochrome *b*_H_ ligands in the PetB protein of acidophilic *Acetobacteraceae* (Fig. S1B) would produce a linear electron transfer equivalent to that carried out by the antimycin-inhibited *bc*_1_ complex, with electrons flowing to cytochrome *c*_1_, which has an intact structure in acidophilic *Acetobacteraceae* (Fig. S10). This situation would resemble, in principle, that described for a mutant of chloroplast cytochrome *b*_6_ which lacks a ligand of cytochrome *b*_H_ but partially sustains photosynthetic electron flow (84). However, in acidophilic taxa, the mutations of cytochrome *b*_H_ ligands are always associated with those of the ISP (Fig. S1 and S9), thereby preventing “back door” pathways for ubiquinol oxidation (84). Much less speculative is the possibility that the Cyc1 and Cyc2 proteins present in acidophilic and photosynthetic *Acetobacteraceae* (Fig. S4) constitute an alternative pathway to reduce *c*-cytochromes in the periplasm, which in turn are re-oxidized by COX as in acidithiobacilli (19) (Fig. 8B). Therefore, the oxidation of ferrous iron, or perhaps other reduced metals, provides the physiological reason why a functional COX is maintained in acidophilic *Acetobacteraceae* that have a deranged *bc*_1_ complex (Fig. S4).

### Conclusions

This work provides a comprehensive and updated analysis of the phylogeny of *Acetobacteraceae*, a large group of alphaproteobacteria that is in need of taxonomic revision. Our data support the rank elevation to the order Acetobacterales proposed previously (28) and indicate two new subdivisions to be added to the order: *Roseomonadaceae*, comprising the *Roseomonas* clade (Fig. 1 and 2; Table 1), and *Acidocellaceae*, encompassing the acidophilic taxa of *Acidisoma*, *Acidocella,* and *Acidiphilium*. The first conclusion emerging from our work is that the latter family is a sister to the acetous clade, which may be re-classified as the family *Acetobacteraceae sensu stricto*. This family could include not only the acetous taxa that originally defined the family (2 – 4) but also the non-acetous genera *Entomobacter*, *Commensalibacter,* and *Asaia* (59). The *Lichenicoccus* group that we have uncovered here (Fig. 1A, 2A, and 3A) forms a subclade that is consistently clustering with the acetous clade and, therefore, could be part of the family *Acetobacteraceae sensu stricto*. This taxonomic proposal would strengthen the concept that all Acetobacterales originated from a photosynthetic ancestor, a novel major conclusion of our work.

Phylogenomic analyses have shown that several Acetobacterales maintain a “historical genomic record” in the form of progressively deranged proteins (especially of the *bc*_1_ complex) and gradual loss of photosynthetic and bioenergetic traits (Table S2 and Fig. S4). Indeed, the differential loss of photosynthetic traits constitutes a trail linking together various clades of Acetobacterales, explaining the increased presence of PQQ-dependent Q reductases as potential compensation for the loss of the Q reductase function of RC. Pathways for oxidoreduction of external metals constitute essential traits in early branching and acidophilic Acetobacterales, providing a functional bypass for the deranged *bc*_1_ complex that such taxa often possess (Fig. 8B). Altogether, our results complete the evolutionary panorama of Acetobacterales showing a progressive transition from versatile photoferrotrophy to the incomplete oxidation of organic substrates defining acetous ecophysiology.

#### Taxonomic proposals

The order Acetobacterales (order cf. reference 28) encompasses the description of the previous family *Acetobacteraceae* by Hördt et al. (3), with the following modification. The order includes three families (*Acetobacteraceae sensu stricto*, *Acidocellaceae,* and *Roseomonadaceae*) and separate genera such as *Elioraea*, *Rhodovastum*, *Rhodopila*, *Acidibrevibacterium,* and *Acidisphaera*.

##### Emended description of *Acetobacteraceae sensu stricto*

The description of the family is the same as that reported by Hördt et al. (3), with the following modifications. This family encompasses all the acetous genera plus *Asaia*, *Commensalibacter,* and *Entomobacter*. It also includes the genera *Endobacter* and *Granulibacter*, as well as *Lichenicoccus* (21) and its group. The majority of the members of the family characteristically possess the physiology of incomplete oxidation of sugars and alcohols, lacking mitochondrial-type cytochrome oxidase. However, some members do have both traits.

##### Description of *Acidocellaceae* fam. nov.

A.ci.do-cel.la´ce.ae. (N.L. fem. n. *Acidocella* type genus of the family; -aceae ending to denote family; N.L. fem. pl. n. *Acidocellaceae*, the family of the genus *Acidocella*). The description of *Acidocella* is essentially that of Wichlacz et al. (85) and Kishimoto et al. (86). This new family includes three genera of strongly acidophilic bacteria: *Acidocella*, *Acidisoma*, and *Acidiphilium*. Their early branching taxa have photosynthetic traits. The family is characterized by deranged forms of the *bc*_1_ complex. The type genus is *Acidocella,* and the type species is *Acidocella facilis* (86).

##### Description of *Roseomonadaceae* fam. nov.

Roz.e.o.mo.na.da´ce.ae. (N.L. fem. n. *Roseomonas* type genus of the family; -aceae ending to denote family; N.L. fem. pl. n. *Roseomonadaceae*, the family of the genus *Roseomonas*). The description is that of Rihs et al. (87). This new family encompasses various genera that either possess or lack photosynthetic traits. The strictly photosynthetic genera are as follows: *Belnapia, Dankookia, Paracraurococcus, Roseicella, Roseococcus, Rubritepida,* and *Sediminicoccus*. The genera *Roseomonas* and *Falsiroseomonas* (1) include both photosynthetic and non-photosynthetic taxa. *Crenalkalicoccus, Caldovatus, Humitalea, Rhodovarius,* and *Siccirubricoccus* do not have photosynthetic traits. The type genus is *Roseomonas,* and the type species is *Roseomonas gilardii* (87).

## Data Availability

The genome of *Acidocella* MX-AZ03 has been deposited in GenBank under the accession CP110774 (GCA_027626035.1). The Whole Genome Shotgun project has been deposited at DDBJ/ENA/GenBank under the BioProject accession number PRJNA914835. The version described here is the first version. MAG sequences presented in this work were deposited at GenBank under the study accession numbers JAQNCT01, JAQNCU01, and JAQNCV01. Additional information pertaining to new and previously reported genomes of Acetobacteraceae is listed in Extended Datasheet 1 posted in the repository https://osf.io/y6gxt/ associated with the manuscript. We have also uploaded the alignments for the trees in Fig. 1A and 2A in FASTA format.

## SUPPLEMENTAL MATERIAL

Supplemental material spectrum.00575-23-s0001.pdf This supplemental material file contains 11 supplemental figures and 2 supplemental tables to complement the data in the manuscript.

ASM does not own the copyrights to Supplemental Material that may be linked to, or accessed through, an article. The authors have granted ASM a non-exclusive, world-wide license to publish the Supplemental Material files. Please contact the corresponding author directly for reuse.
