# Supplementary material for: The phylogeny of Acetobacteraceae: photosynthetic traits and deranged respiratory enzymes
Source: Microbiol Spectr. 2023 Nov 17;11(6):e00575-23. doi: 10.1128/spectrum.00575-23 (PMC10715153; doi:10.1128/spectrum.00575-23)
Supplement: Supplemental material — This supplemental material file contains 11 supplemental figures and 2 supplemental tables to complement the data in the manuscript. [file spectrum.00575-23-s0001.pdf]

The phylogeny of Acetobacteraceae: photosynthetic traits and deranged respiratory enzymes

Supplemental Material

This paper is supported by a total of 11 Supplementary Figures, two Supplementary Tables and four additional References, which are listed below following the numerical sequence of those in the main text.

Additional References

[88] Tavaré, S. Some probabilistic and statistical problems in the analysis of DNA sequences. Lectures on mathematics in the life sciences. 1986; 17(2), 57-86. No DOI available.

[89] Hirsch P, Hoffman B. Dichotomicrobium thermohalophilum, gen. nov., spec. nov., budding prosthecate bacteria from the solar lake (Sinai) and some related strains. Syst. Appl. Microbiol. 1989; https://doi.org/10.1016/S0723-2020(89)80027-X.

[90] Marasco, E. K., & Schmidt-Dannert, C. Identification of bacterial carotenoid cleavage dioxygenase homologues that cleave the interphenyl α, β double bond of stilbene derivatives via a monooxygenase reaction. ChemBioChem. 2008; https://doi.org/10.1002/cbic.200700724.

[91] Kawakami T, Yu LJ, Liang T, Okazaki K, Madigan MT, Kimura Y, Wang-Otomo ZY. Crystal structure of a photosynthetic LH1-RC in complex with its electron donor HiPIP. Nat Commun. 2021; https://doi.org/10.1038/s41467-021-21397-9.

Supplementary Figure S1. Expanded phylogeny of Acetobacteraceae. A. The 16S rRNA of at least two representative taxa for most currently recognized genera of the family Acetobacteraceae [2,4] (see also https://lpsn.dsmz.de/family/acetobacteraceae, accessed on 1 November 2022) was retrieved and aligned with MUSCLE v3.8.1551. The final alignment contained 78 sequences and 1467 bases and was refined as described earlier [88]. The best fit GTR model [82,83] was then used with the parameters -st DNA -m GTR+I+G4+FO -bb 1000 -alrt 1000 to reconstruct a reproducible ML tree using the web version of the IQ-Tree program [82]. The numbers indicate the % of Ultrafast bootstrap support of the nodes [82]. **B.** Alignment bloc of the fourth transmembrane helix (TM4) of cytochrome *b* which binds both hemes of the protein. The numbering of yeast cytochrome *b* [65] is shown at the bottom of the alignment: His182 is the axial ligand for the low potential cytochrome *b*, b_L, while Hist197 is the axial ligand for the high potential cytochrome *b*, b_H, which directly interacts with Q together with His202 [65], indicated by Q in yellow highlight on top of the alignment. The conservative substitution of His202 with Arg is present in acetous *Entomobacter* and many acidophilic taxa (highlighted in pale blue) as in chloroplast cytochrome b_6, while the other His ligand of b_H is substituted not conservatively in most *Acidiphilium* and *Acidocella* taxa [65]. **C.** The ML phylogenetic tree of cytochrome *b* of Acetobacteraceae was reconstructed with 78 sequences as in A, compensating for taxa that do not have cytochrome *b* (*Granulibacter*, *Asaia bogoriensis*, *Acidomonas* and *Swingsia*) with proteins from closely related taxa. The alignment had 445 amino-acid sites, 4.05% of which were constant, and the tree was reconstructed with the LG model, found to be the best model as described earlier [83]. Note the strong support (97% of Ultrafast bootstraps [82]) for the node linking the sister clades of acidophilic and acetous taxa. The clade labelled ‘other photosynthetic’ included *Rhodopila*, *Acidibrevibacterium* and three different *Acidisphaera* taxa.

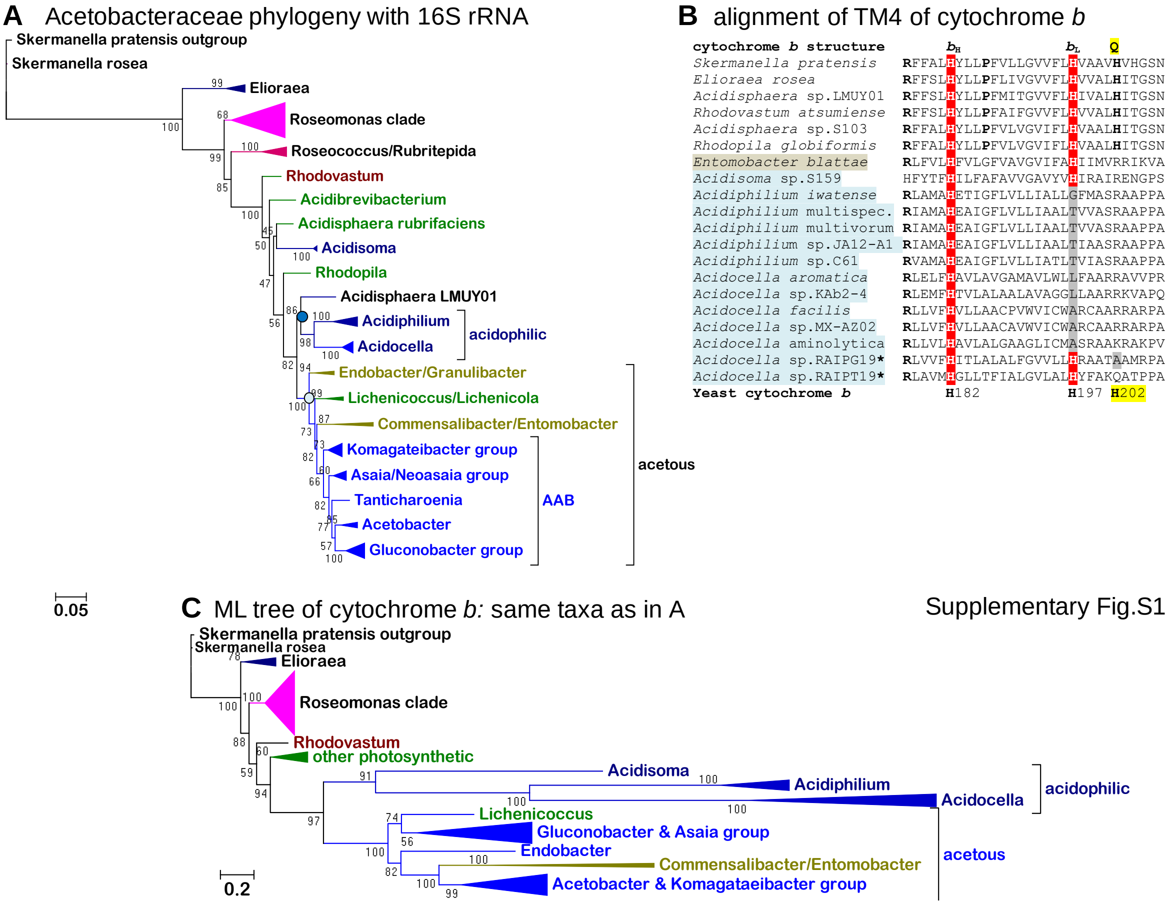

Supplementary Figure S1. D. Expanded phylogeny of Acetobacteraceae using 32 concatenated core proteins. The ML tree was reconstructed with IQ-Tree [82] from a concatenated alignment comprising the same 15 ribosomal proteins used in Fig. 1A plus 17 core proteins that were present in at least 56 of the 58 taxa used to represent Acetobacteraceae groups and the *Skermanella* outgroup. The additional core proteins, found using GTDB-Tk software, (see Expended Datasheet 2 in repository https://osf.io/y6gxt/ for details) were: RimP N-terminal domain; 16S rRNA (cytosine(1402)-N(4))-methyltransferase; phenylalanyl-tRNA synthetase, alpha subunit; ribosome recycling factor; transcription termination/antitermination factor NusG; DNA polymerase III, delta subunit; inosine-5'-monophosphate dehydrogenase; excinuclease ABC, C subunit; leucyl-tRNA synthetase; methionyl-tRNA synthetase; DNA repair protein RadA; GTP-binding protein Era; phospho-N-acetylmuramoyl-pentapeptide-transferase; Holliday junction DNA helicase, RuvB subunit; DNA-directed RNA polymerase, alpha subunit; DNA-directed RNA polymerase, beta' subunit; and CTP synthase. The alignment had 10198 amino-acid sites, 35.86% of which were constant. The tree was reconstructed with the LG model found to be the best [83] as in Fig. 1A. Note that the branching pattern and statistical support values of major nodes were similar to those of the ML tree obtained with only the concatenated ribosomal proteins (cf. Fig. 1A).

D ML tree of 32 concatenated core proteins including 15 ribosomal

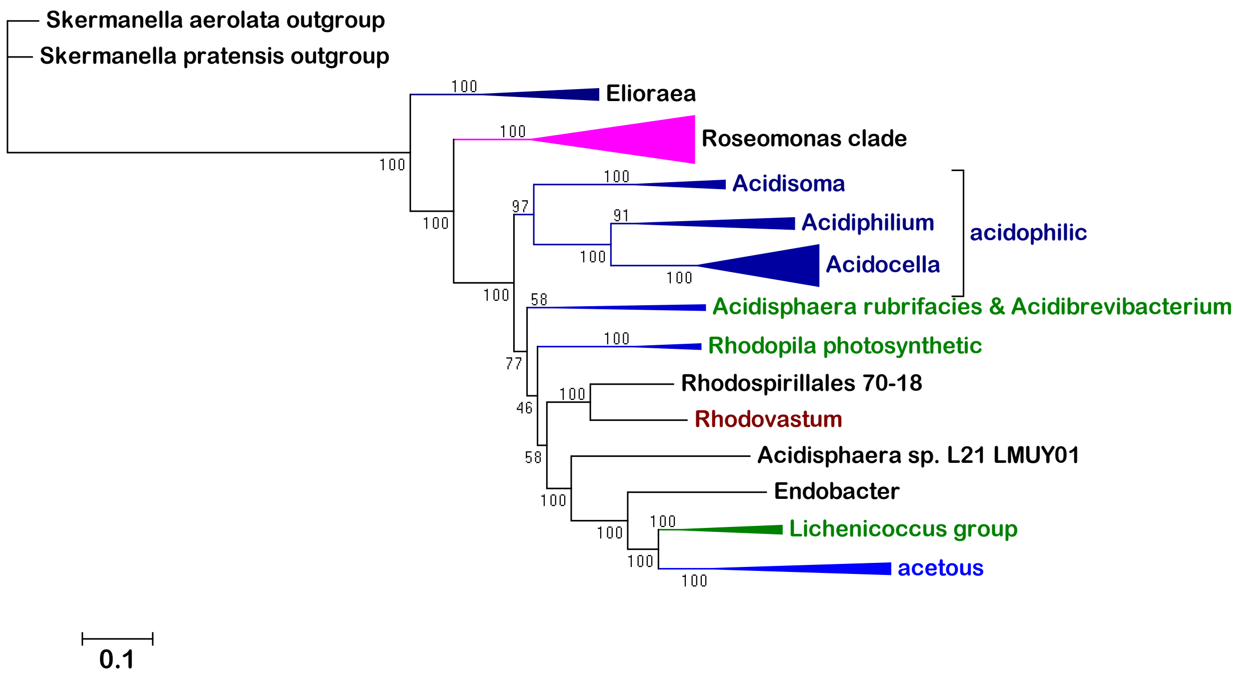

Supplementary Fig. S2. Phylogenetic distribution of COX1 proteins of type A1 subtype b in Acetobacteraceae. **A.** The dot plot shows the distribution of cytochrome *c* oxidases vs. that of photosynthetic RC. COX A2 indicates A2 type, subtype a-I HCO A family [20]; ba3 indicates HCO B family subtype ba3 [20]; cperox indicates cytochrome *c* peroxidase. Note that a ba3 oxidase is present also in acetous *Acidomonas methanolica* [58], in which it has not been recognized before as a B family oxidase. **B.** ML tree reconstructed with a manually curated alignment of a selection of 74 COX1 proteins, which included 592 amino-acid sites (21.6% of which constant), obtained with the best-fit model [83] LG and gamma = 4. *Zavarzinia* COX1 used as an additional outgroup is cut off.
C. Phylogenetic tree of COX3 subunit. The ML tree was reconstructed with 65 sequences of COX3 from subtype b COX operon containing 326 amino-acid sites, 27.6% of which were constant, and using the EX_EHO model. Note the clustering of one Antarctic MAG with *Siccirubricoccus* within the *Roseomonas* clade (cf. Table 2).

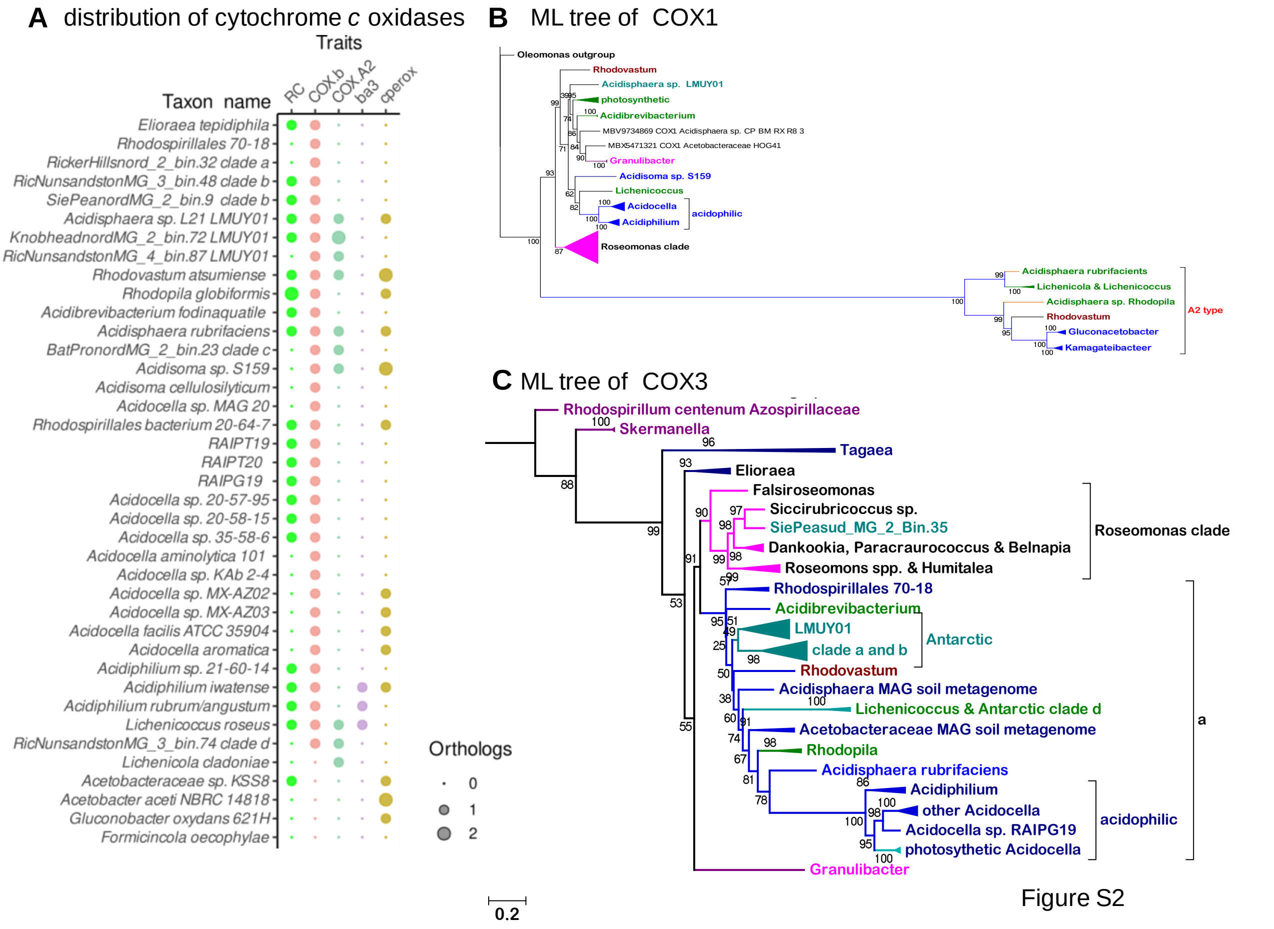

Figure S2

Supplementary Figure S3. **Distribution of photosynthetic and ChlB proteins. A.** The NJ tree of PufM proteins is an expansion of that shown in Fig. 4A. The large clade containing all other protein hits has been cut-off completely. Note that the Acetobacteraceae clade includes protein hits from a few other alphaproteobacteria, in particular Azospirillaceae (*Skermanella* and *Rhodospirillum centenum*) and the genus *Salinarimonas* of the Rhizobiales order. The gray arrow indicates the position of proteins from three Deltaproteobacteria MAGs and one Gemmatimonadetes MAG which have been removed, because the focus was on alpha-, beta- and gamma-proteobacteria as in previous studies [44]. These hits were likely due to LGT. The symbols indicate photosynthetic physiology as in Figs. 3 and 4. It is interesting to note the upstream position of the PufM of *Dichotomicrobium thermohalophilum* [89] in the branch subtending position of the Acetobacteraceae clade, which includes only one other likely AAP, *Algihabitans*. The outgroup position of *Caenispirillum salinarum* PufM resulted from default parameters of distance tree reconstruction in BLAST routine. The protein is not basal in the PufM family but appears to derive from LGT with photosynthetic Rhizobiales such as *Fulvimarina* as its possible source. **B.** Phylogenetic tree of PufC, the tetraheme cytochrome *c* subunit of the RC. This protein is the entry point for electrons from either HiPIP or soluble *c* cytochromes, mainly cytochrome c_2 homologs, to re-reduce the photo-oxidized special pair of chlorophyll in the RC [44]. In several bacteria with anaerobic photosynthetic physiology, PufC contains a lipid anchor that is attached to a Cys residue following a hydrophobic stretch at the N terminus, which forms a transmembrane helix in *Rhodopila* and most AAP [54]. Only a few members of the *Roseomonas* clade maintain this Cys residue. The ML tree was reconstructed from an alignment of 50 sequences with 406 amino-acid sites, 12.4% of which were constant, with the best fit model [83] WAG. Note how PufC of *Rhodovastum* and *Rhodopila* cluster with those of acidophilic Acetobacteraceae, in contrast with PufM (Fig. 4A) and PufL subunit. This result was confirmed using other substitution models and was in agreement with the phylogeny of other photosynthetic markers (part C). **C.** Phylogenetic tree of ChlB, protochlorophyllide:ferredoxin oxidoreductase. This protein is an essential part of the system for the biosynthesis of bacteriochlorophyll and a useful phylogenetic marker [44]. The ML tree was reconstructed from an alignment of 84 ChlB sequences with 570 amino-acid sites, 31% of which were constant, using the best fit model [83] LG. *Caenispirillum* ChlB has been used as outgroup to match the phylogenetic tree in part A.

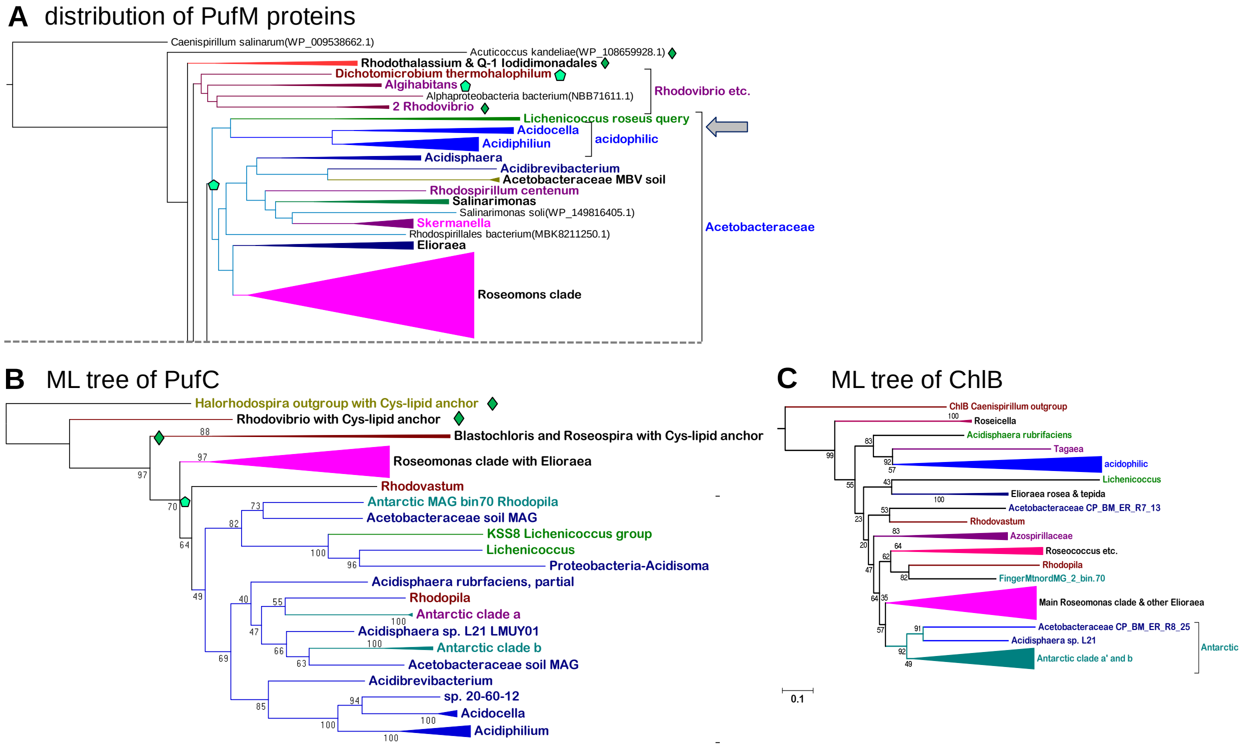

Figure S3

Supplementary Fig. S4. Distribution of photosynthesis-related traits vs. iron-oxidizing cytochromes and ubiquinol oxidoreductases. This list encompasses various traits coded by genes that are generally associated with the photosynthetic gene cluster including the PufLMC subunit of the RC [50], in particular the Crt coding genes for the biosynthesis of carotenoids. These genes are subdivided in two groups: CrtBCEF and CrtIfam, the latter including both CrtD and CrtI carrying out similar desaturase reactions, which have homologs also in non-photosynthetic Acetobacteraceae such as *Acidomonas*. The list includes two additional enzymes for carotenoid metabolism: Cco, representing carotenoid cleavage oxidases such of the REP85 super-family that split carotenoids into smaller metabolites [90], and β-Car representing beta-carotene oxygenases that produce retinal, often associated with the bacteriorhodopsin gene [57], as indicated in the opsin column. The list includes Form I of Rubisco (abbreviated as RbcL), which in *Acidomonas* (#) is associated with methylotrophy [52]. The electron donors to the RC, HiPIP (Hip) and soluble cytochrome c_2 (c2), are listed aside the leftmost PufMC column containing the RC subunits. The absence of c_2 is indicated by a "no", while its substitution by a di-heme c_2 is indicated by **. The right side of the list presents the distribution of active (red) and deranged (dark gray) bc_1 complex, as well as that of Cyc2, the entry point of electrons from external iron and its redox partner Cyc1[70,73]. On the far right are the two types of *bd* ubiquinol oxidase [16] and the bo_3 ubiquinol oxidase. Taxa are expanded with respect to those in Supplementary Table S2 and Fig. 5A to include photosynthetic species of the *Roseomonas* clade (cf. Table 1) and a few acetous species retaining carotenoid genes.

taxon name	PufMC	Hip	c2*	CrtBCEF	CrtI fam	Cco	β-Car	opsin	RbcL	bc1	Cyc2	Cyc1	bd-I	Cio	bo3		COX A1 b	COX A2 a-I	B ba3
Elioraea tepidiphila			2																
Roseomonas nepalensis			2					1											
Falsiroseomonas bella			3																
Dankookia rubra			4						1										
Paracraurococcus ruber		2	6																
Belnapia mucosa			2																
**Rhodospirillales 70-18**			4																
**RickerHillsnord_2_bin.32 clade a**			2																
**RicNunsandstonMG_3_bin48 clade b**	2	2	2					**4**	1										
**SiePeanordMG_2_Bin.9 cade b**			2					1	1					2					
***Acidisphaera* sp. L21 LMUY01**			no											2	2				
**KnobheadnordMG_2_bin.72 LMUY01**			1																
**RicNunsandstonMG_4_bin.87 LMUY01**			no															2	
Rhodopila globiformis	2	**4**	6						1		3								
Rhodovastum atsumiense			4						1		2								
Acidisphaera rubrifaciens		2	4						1										
Acidibrevibacterium fodinaquatile		2	2						2			2							
**BatPronordMG_2_bin.23 clade c**			2																
**Proteobacteria G5_19** *Acidisoma*			1												**3**				
Acidisoma sp. S159			1									3							
***Acidisoma* sp. L85**			1									3			2		split operon		
Acidocella sp. MAG 20			1																
Rhodospirillales 20-64-7 *Acidocella*			1																
**Acidocella sp. RAIPT19**		3	1						1										
**Acidocella sp. RAIPT20**			1						1										
Acidocella sp. 20-57-95			1																
Acidocella sp. 20-58-15			2																
Acidocella sp. 35-58-6			**																
Acidocella aminolytica 101		2	**																
Acidocella sp. KAb 2-4			2																
**Acidocella sp. MX-AZ03**			**												2				
Acidocella facilis ATCC 35904			**											2	2				
Acidocella aromatica			**																
Acidiphilium iwatense			1						1										
Acidiphilium rubrum/angustum		2	no						1										
**RicNunsandstonMG_3_bin.74 clade d**			1						1			3		**3**	2				
**Acetobacteraceae sp. KSS8**			**												2				
Lichenicoccus roseus			1^					1							**3**				
Lichenicola cladoniae			**					1				2			**3.5**				
Endobacter medicaginis			1			**6**													
Acidomonas methanolica			no			3			2#										
Gluconacetobacter diazotrophicus			1																
Acetobacter aceti NBRC 14818			1											2					
Gluconobacter oxydans 621H			no																
Formicincola oecophyllae			no																

Supplementary Fig. S5. Molecular interaction of the C terminus of pufM with the heme 3 region of pufC.
A. 3D structure of *Rhodopila* PufM (bluish) and PufC (red) from Ref [54] rendered with iCn3D Structure Viewer https://www.ncbi.nlm.nih.gov/Structure/icn3d accessed on 10 December 2022. The square symbol indicates the approximate position of heme 3 in PufC [54], while the round blue symbol labelled Hip indicates the approximate binding position of mature HiPIP with the region of PufC containing heme 1, the first electron acceptor in RC [91]. The regions of PufM that interact with PufC are in darker blue to purple. **B.** The alignment shows the C-terminal region of a selection of PufM proteins from taxa that have anaerobic phototrophic physiology and lack PufC (first two lines) and other that have the PufC protein, including *Rhodopila* (underlined) and several representatives of the Acetobacteraceae family. The boxed residue corresponding to the C terminus of *R.rubrum* PufM lies at the beginning of the last region interacting with the PufC part surrounding heme 3 [54], as shown in part A (arrow).

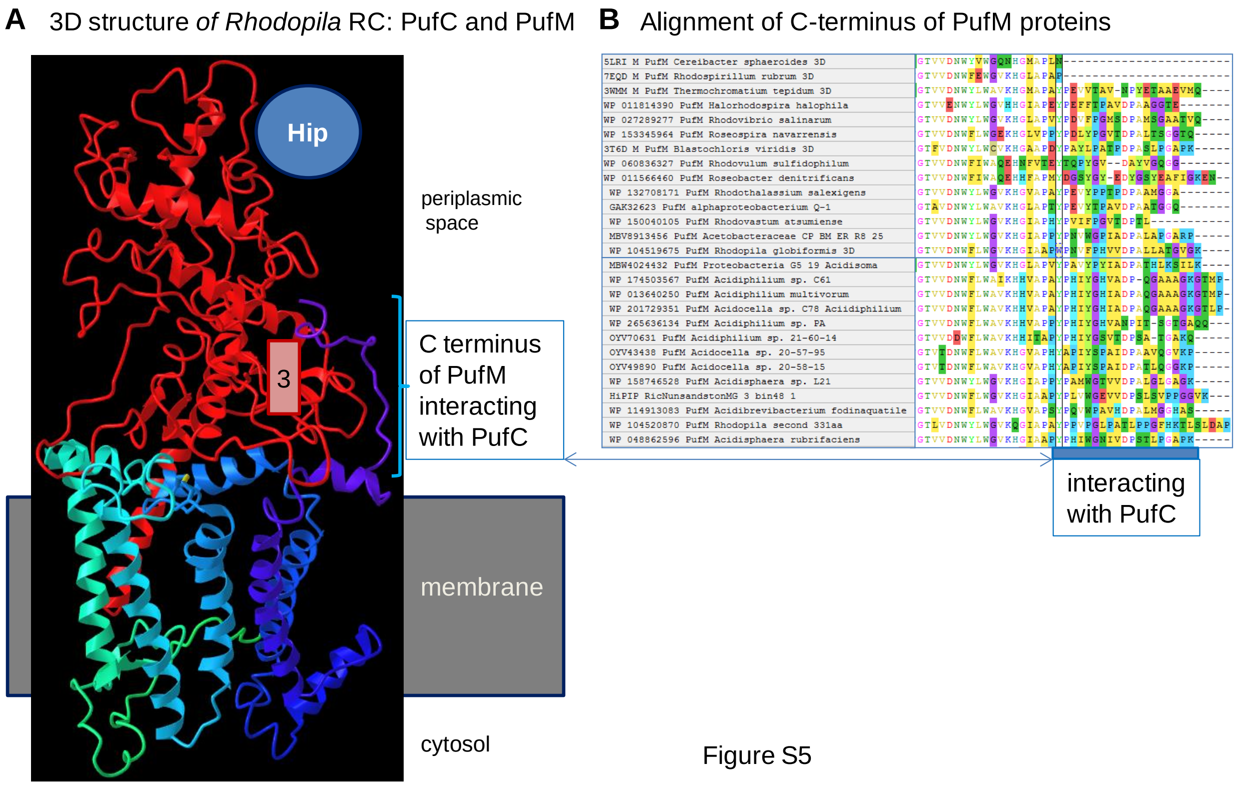

Figure S5

Supplementary Fig. S6. Phylogeny of the CyoA subunit of *bo*$_3$ ubiquinol oxidase. A. The NJ tree was obtained from a PSI-BLAST1000 of CyoA from *Acidithiobacillus ferrivorans* retaining a Cys ligand for Cu$_A$ [19] against all proteobacteria. Proteins shorter than 250 amino acids (partial or fragments) were excluded from the selection used to build the tree. Note that the early branching position of a few *Legionella* and *Salinisphaera* spp. likely derive from tree artifacts due to their long branch. Clade 1 of acidophilic taxa is indicated by the numeral. **B.** The NJ tree was obtained from a PSI-BLAST1000 of CyoA AWP22960 from *Acidiferrobacter* sp. as a query against all alphaproteobacteria, Acidithiobacillia and *Acidiferrobacter thiooxydans*. The very large clade including all other proteins from alphaproteobacteria (gray) is cut off. Dashed arrows show the correspondence with the clades in A. **C.** The ML tree was reconstructed with IQ-Tree [82] from an alignment of 167 CyoA proteins and 3 COX2 paralogs and manually refined taking into consideration also known structures; this alignment had a total of 412 amino-acid sites, 2.67% of which were constant. The tree was obtained with the mixture model EX_EHO as in Fig. 6B and rooted midpoint.

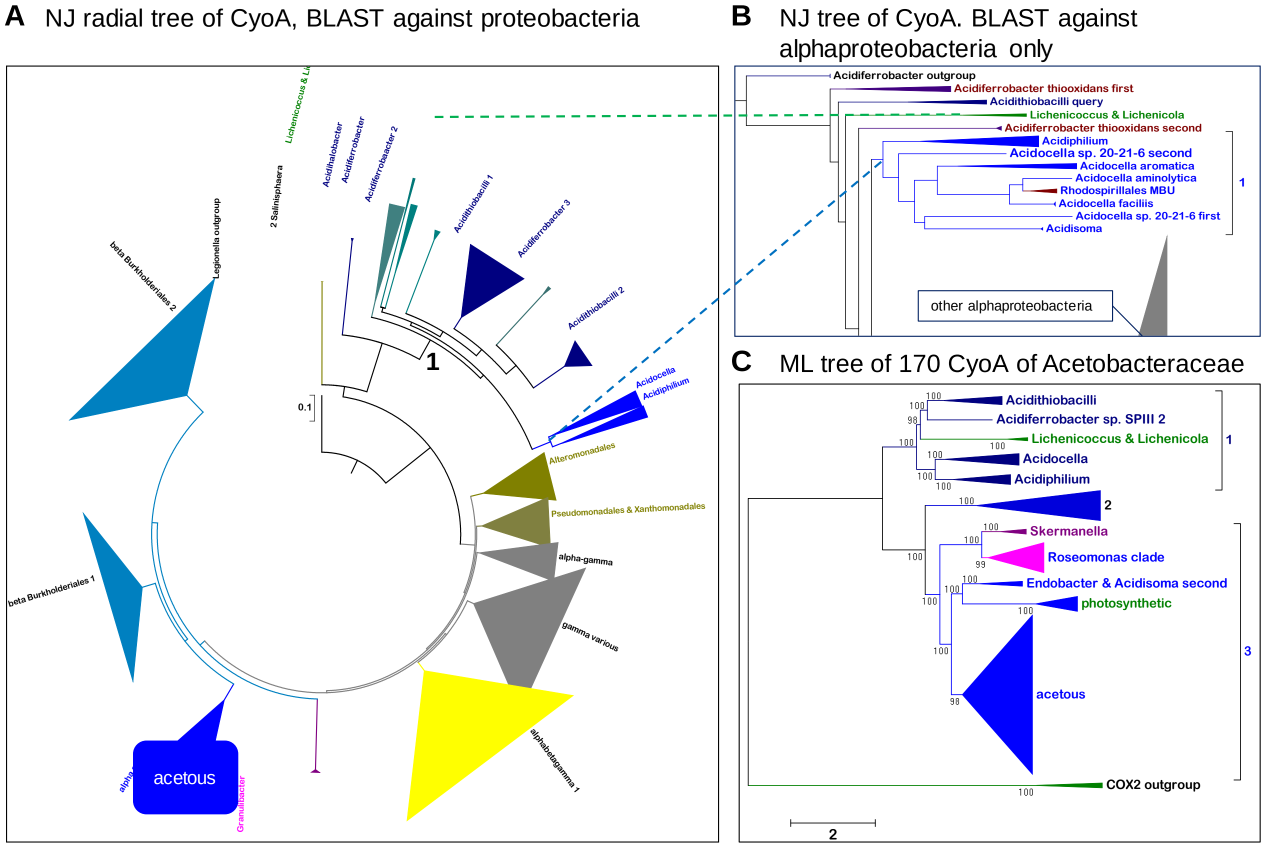

Figure S6

Supplementary Fig. S7. Phylogeny of the CyoB large catalytic subunit of *bo*$_3$ ubiquinol oxidase. A. The ML tree was obtained with the mixture model EX_EHO from an alignment of 130 CyoB sequences very similar to that for the ML tree in Fig. 6B. The alignment included 732 amino-acid sites, 96 of which were constant.

B. NJ tree obtained from a PSI-BLAST of *Roseomonas cervicalis* CyoB against all genera of the *Roseomonas* clade (*Roseomonadaceae*) in Table 2, plus *Lichenicoccus* and *Acetobacter tropicalis*, which cluster with the same clade (Fig. 6B). All taxa different from the dominant *Roseomonas* spp. are in fucsia, while two of the three *Lichenicoccus* cyoB are in green as in Fig. 6. The pink box corresponds to the *Roseomonas* clade in A as indicated by the arrow. Note that the *bo*$_3$ oxidase is present only in five out of the 14 genera of the *Roseomonas* clade shown in Table 2, and 29 out of 77 *Roseomonas* taxa that are currently available in the nr database (accessed on 24 Nov 2022).

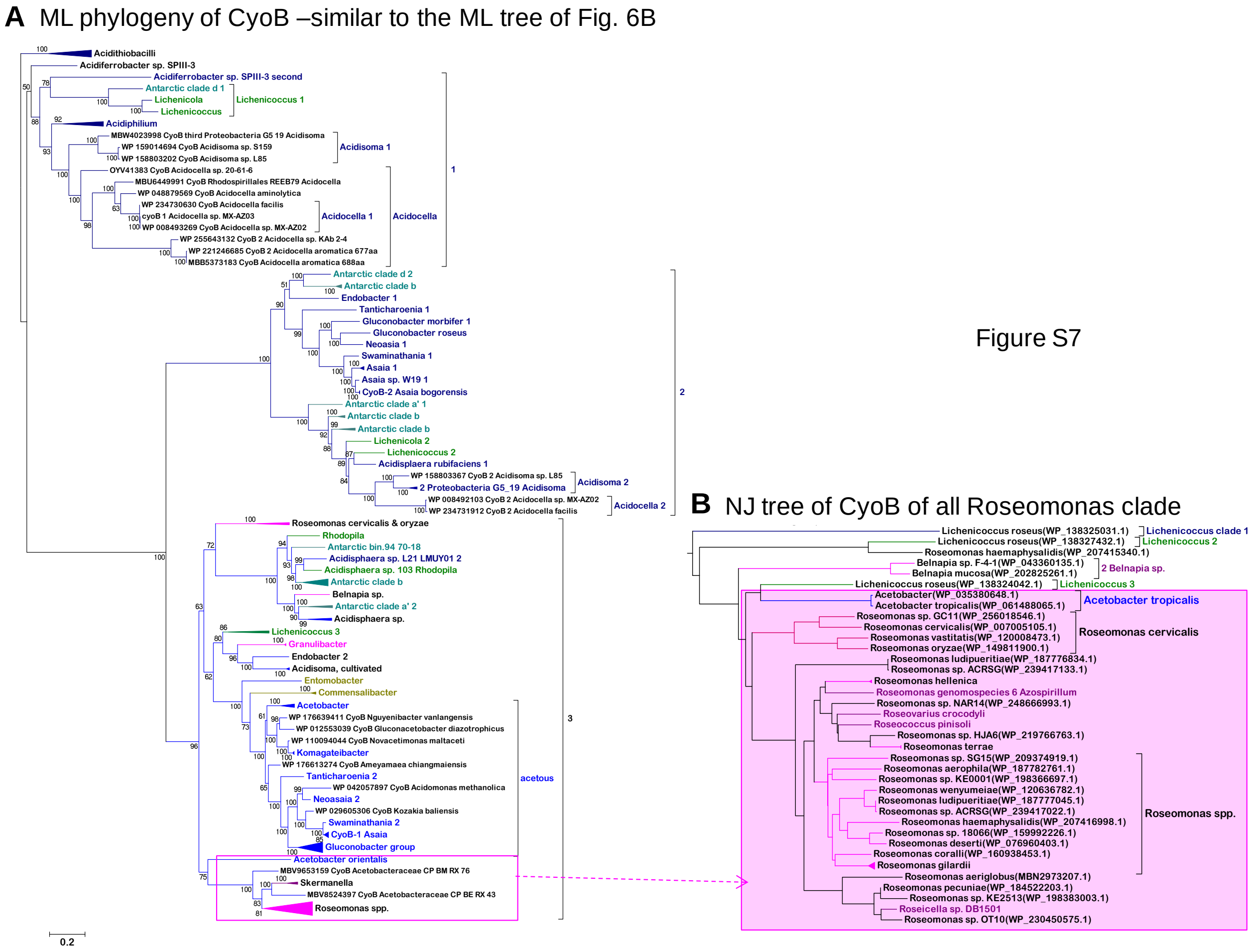

Supplementary Fig. S8. A. Variation in the PetA Rieske ISP proteins of the *bc*$_1$ complex in Acetobacteraceae. **B.** Structure of the ISP cluster and its ligands, two cysteines and two histidines [66]. **C.** The alignment bloc encompasses the C-terminal region binding the ISP cluster of the PetA subunit of the *bc*$_1$ complex in selected Acetobacteraceae and shows the molecular variation of the ligand structure in acidophilic taxa. The alignment was rendered using the MEGA X program with residues toggled at 80% conservation. Unclassified Acidisoma corresponds to *Acidisoma* sp. S159, the only *Acidisoma* taxon possessing genes for the *bc*$_1$ complex (Supplementary Table S2). Fully conserved residues are indicated by asterisks on top of the alignment. The ligand residues are represented by the yellow arrows, while the position of Tyr165 regulating the redox potential of the ISP protein [67,68] is boxed and indicated by the blue arrow. Cimit5 identifies the insert of several residues separating the two ligand signatures in the ISP protein of various proteobacteria [65]. Notably, Cimit5 is present only in representatives of the *Lichenicoccus* group at the bottom of the alignment, separated by the continuous line. The long form of ISP of acetous taxa also shows the Cimit5 insert [65,69].

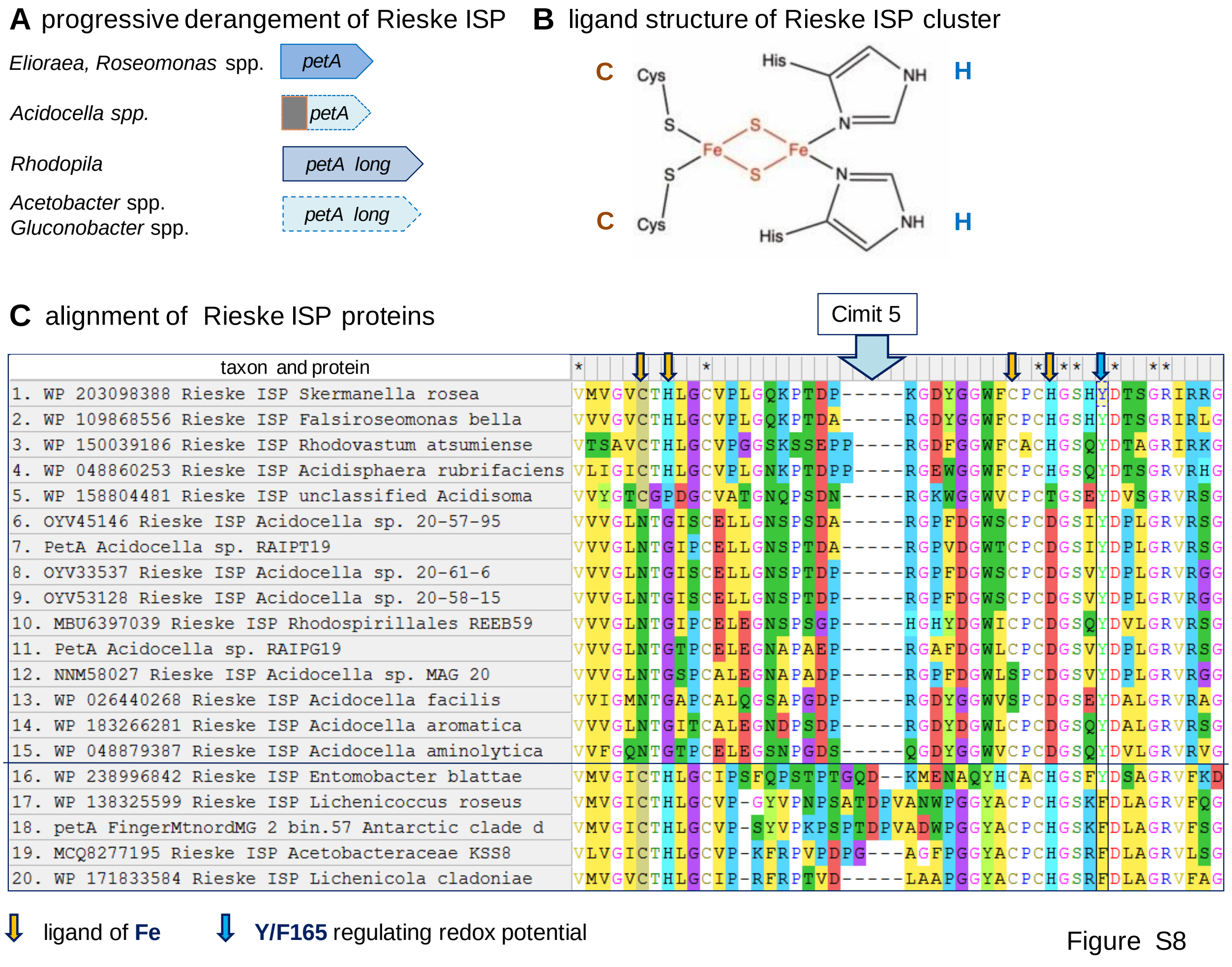

Supplementary Fig. S9. Phylogeny of the ISP subunit of the *bc_1* complex. The phylogenetic ML tree of 130 Rieske ISP proteins of representative Acetobacteraceae was obtained with the WAG model. The arrow with LGT indicates the probable insertion of long ISP proteins from beta- and gamma-proteobacteria into Acetobacteraceae lineages [65], as in Fig. 7B. See Supplementary Fig. S8 for complementary information.

phylogeny of the ISP subunit

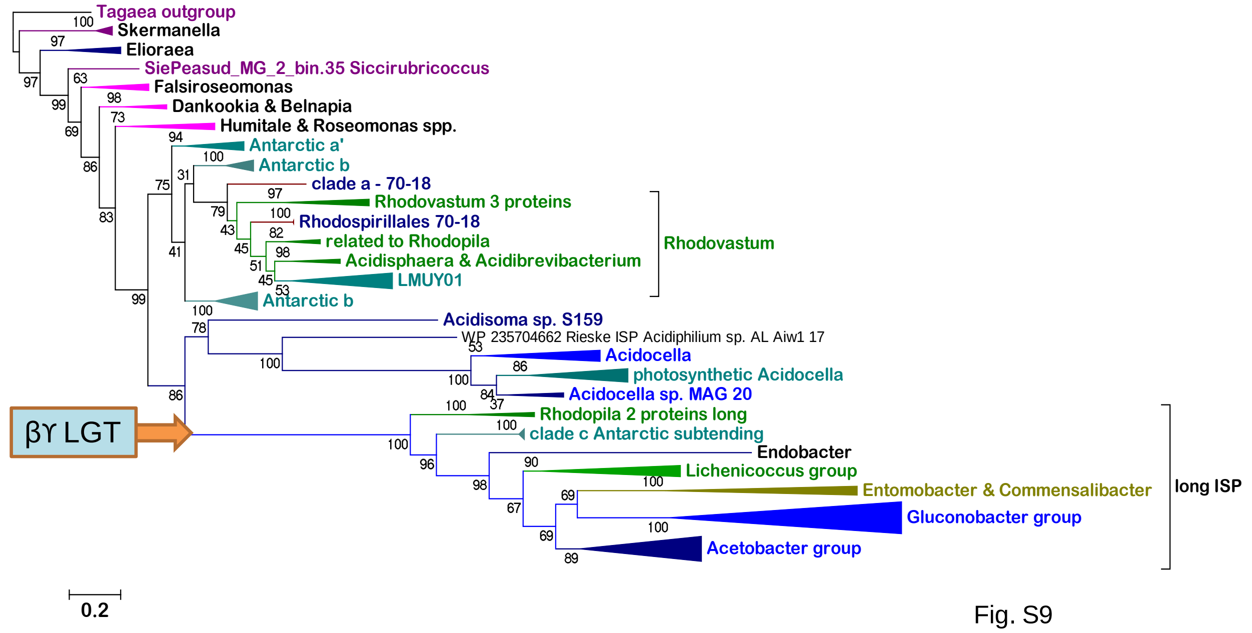

Fig. S9

Supplementary Fig. S10. Phylogeny and structure of PetC cytochrome c_1 among Acetobacteraceae. A. The ML tree was obtained from an alignment of 42 cytochrome c_1 sequences with 291 amino-acid sites, 9% of which were constant and included all the conserved signatures for heme binding [76,78]: CxxCH towards the N terminus and the distal Met in the middle of the protein (indicated in part B). The tree was built with the best model WAG as in Fig. S9. **B.** The alignment block covers the central region of the PetC cytochrome c_1 protein containing the Met distal ligand of the heme, which is boxed and indicated by the red symbol.

A ML phylogeny of cytochrome *c*1

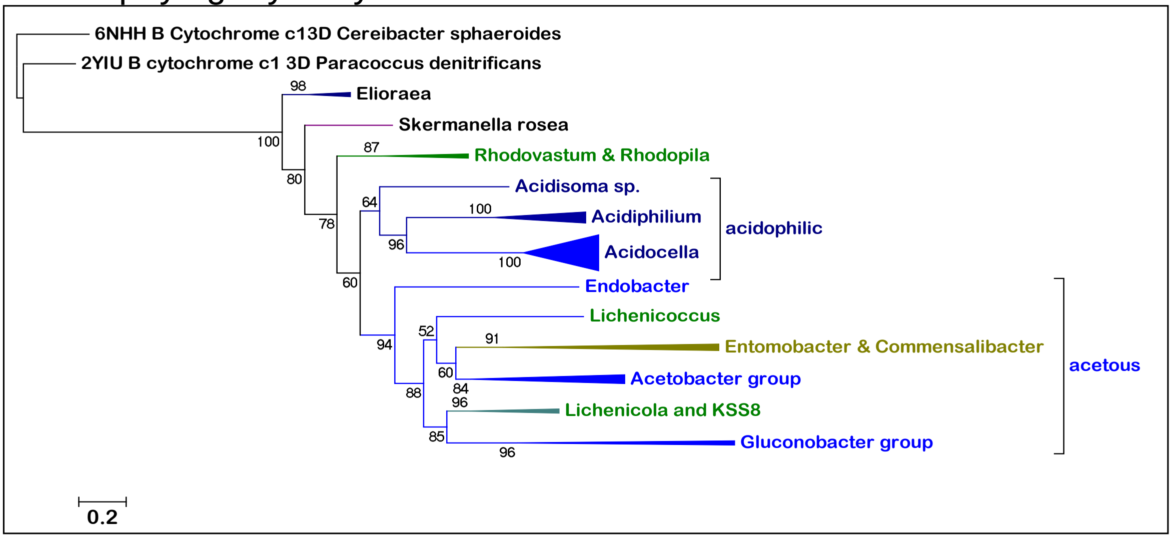

B alignment block of cytochrome *c*1 with the Met ligand

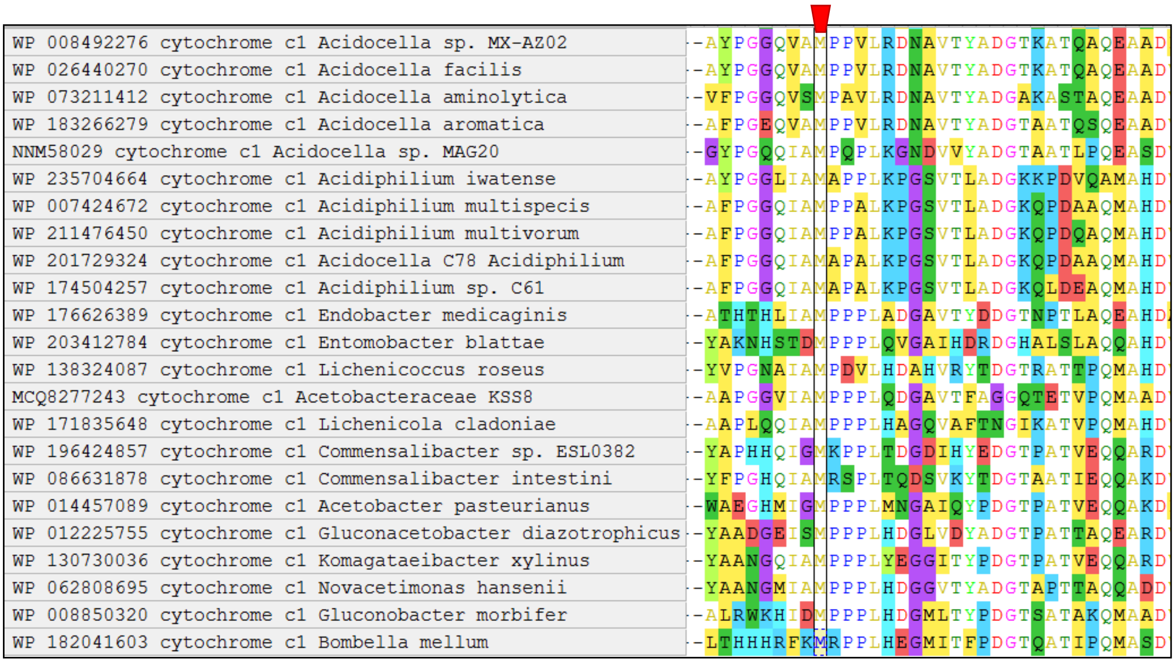

Figure S10

Supplementary Fig. S11. Phylogeny of the Cox15 protein among Acetobacteraceae.
A. The ML tree was reconstructed with 68 sequences of Cox15, heme A synthase, representing all genera that contain the gene for the protein. The tree was reconstructed with a manually curated alignment [33] containing 402 amino-acid sites, 9% of which were constant, and the best-fit model [83] WAG with gamma = 4 as in Supplementary Fig. S9. Note that some *Acidocella* and other acidophilic taxa have two forms of Cox15, one of which is labeled 'deranged' because it does not have the conserved ExxH signature. **B.** The alignment block covers the region containing the conserved ExxH signature that is essential for heme A synthesis [33]. This signature is indicated by the two red arrows and has been lost in several acidophilic taxa.

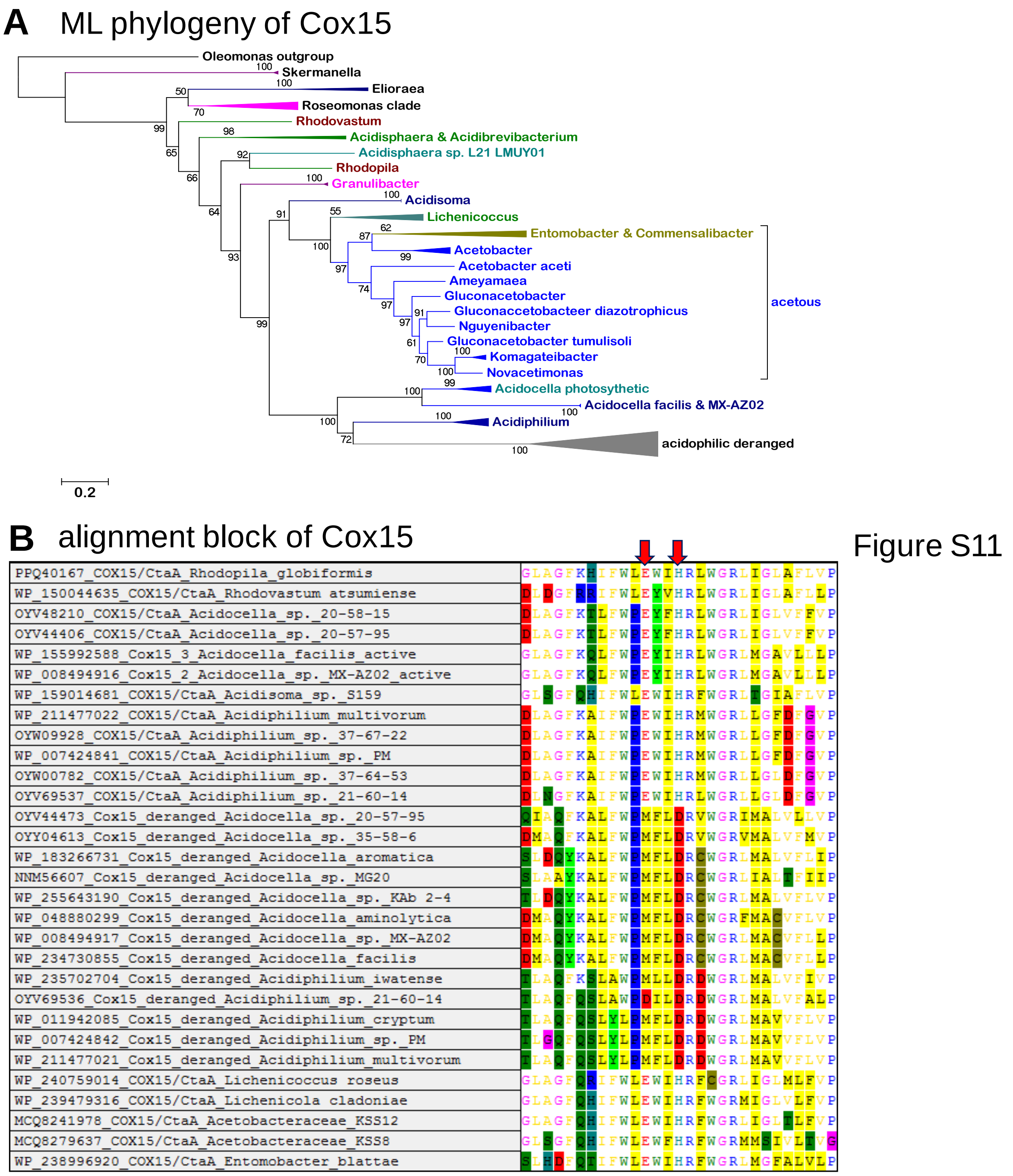

Supplementary Table S1. Genomic characteristics of *Acidocella* and related taxa including the proteobacteria MAG classified as *Acidisoma* (see text). Genome completeness values above 95% are in bold. The new *Acidocella* taxa presented in this work are in blue characters. GTDB classification (https://gtdb.ecogenomic.org/ accessed on 18 May 2023) has been fundamentally used for classifying the taxa. However, taxa indicated by the asterisk* have been (re)classified in this work following the consensus of different phylogenetic trees, as described in the Methods. Additional genomic information on the taxa shown here ,as well as other taxa studied in this work, are presented in Expanded Datasheet 1 posted in the online repository https://osf.io/y6gxt/, which is associated with the manuscript. Taxa are deduced to have photosynthetic traits when their genome includes the *puf* genes of the photosynthetic reaction center. We have analysed the genome of *Acidocella aquatica* from its contigs highlighted in pale green (https://www.ncbi.nlm.nih.gov/datasets/genome/ , accessed on 18 May 2023).

taxon name	accession	classification	GC%	**bold** over 95% completeness	proteins
Acidisphaera sp. L21 LMUY01	GCA_009765685.1	g__LMUY01	64.87	**95.02**	6169
Proteobacteria G5_19 = Acidisoma	GCA_019351895.1	Acidisoma*	66.75	**98.13**	4248
Acidisoma sp. S159	GCA_009766125.1	Acidisoma sp009765865	62.62	**100**	6160
Acidocella aromatica	GCF_014201825.1	Acidocella	63.99	**100**	2736
Acidocella aminolytica 101	GCF_000964385.1	Acidocella	58.94	**98.51**	4154
Acidocella facilis ATCC 35904	GCF_000687875.1	Acidocella	64.51	**100**	3216
Acidocella sp. MX-AZ02	GCF_000306035.1	Acidocella	64.13	**95.74**	3462
**Acidocella sp. MX-AZ03***	GCA_027626035.1	Acidocella	63.66	94.11	3531
**Acidocella sp. KAb 2-4**	GCF_020567705.1	Acidocella sp020567705	65.24	**98.96**	2988
Acidocella sp. MAG 20	GCA_013044125.1	Acidocella sp013044125	63.10	92.45	2688
Acidocella aquatica	BSOS01000001-137	Acidocella sp013044125*	61.75	**99.50**	3769
Acidocella sp. 20-58-15	GCA_002255785.1	Acidocella sp002281005	57.62	76.86	2405
Acidocella sp. 20-57-95	GCA_002255575.1	A. sp002281005	57.07	**98.99**	3017
Acidocella sp. 21-58-7	GCA_002255715.1	A. sp002281005	57.84	92.86	2853
Acidocella sp. 35-58-6	GCA_002281005.1	A. sp002281005	58.44	81.72	2151
**Acidocella sp. RAIPT19***	GCA_029077825.1	A. sp002281005*	56.78	**98.31**	2599
**Acidocella sp. RAIPG19***	GCA_029077775.1	Acidocella sp.	60.52	87.95	3500
**Acidocella sp. RAIPT20***	GCA_029077765.1	Acidocella sp.	57.74	57.9	2533
Rhodospirillales bacterium 20-64-7	GCA_002255195.1	undefined# - Acidocella*	64.28	78.93	5729
Rhodospirillales bacterium REEB79	GCA_018971565.1	Acidocella sp018971565	60.04	84.09	2182
Rhodospirillales bacterium REEB59	GCA_018971905.1	Acidocella sp018971905	61.47	81.46	2183
Proteobacteria bacterium REEB7	GCA_018971725.1	Acidocellasp018971725	61.69	79.81	2452
Acidocella sp. 20-61-6 ^	GCA_002255625.1	undefined# - mixture of 2MAGs, one close to sp013044125*	61.33 average	80.17	3404
Acidocella sp. C78	GCF_907164655.1	undefined# - Acidiphilium*	67.09	91.67	6322
Rhodospirillales bacterium 20-60-12	GCA_002255495.1	g__20-60-12 - Acidiphilium*	59.86	94.08	2825
Acidiphilium sp. 21-60-14	GCA_002255745.1	Acidiphilium	60.2	**99.5**	2828

#undefined by GTDB

^The genome is a mixture; complex I and photosynthetic proteins cluster with photosynthetic Acidocella

*re-classified as recommended in this work

Supplementary Table S2. Distribution of the various bioenergetic traits analyzed in this work among various Acetobacteraceae. The new *Acidocella* taxa presented in this work are in bold blue characters. The number of orthologs defining any given trait is annotated, in bold when equal to three or more. Column headings are as follows, with noted number of genes per trait: Q, ubiquinone biosynthesis (7 genes; TUV, with additional 3 genes for anaerobic biosynthesis); ETF-Q, Electron Transfer Flavoprotein Dehydrogenase Q-reducing (single gene); nuo13 and nuo14, different operons for Complex I (13 or 14 genes); Sdh, succinate-Q reductase or complex II (5 genes); PQQde, PQQ-dependent dehydrogenase (1-3 genes); SoxBY, genes for sulfate reduction [39]; Hyd1, Q-reducing hydrogenase [59] (5 genes); Codh, Carbon monoxide dehydrogenase (*both forms [59], 3 genes); mSor, novel Sulfoxide-Q oxidoreductase (2 genes); RC, photosynthetic Reaction Center (3 genes); bo3, ubiquinol (QH_2) oxidase of the HCO family [19] (4-5 genes); bd-1, cytochrome *bd* ubiquinol oxidase of the bd-I type [16] (3 genes); Cio, cytochrome *bd* ubiquinol oxidase of the CIO type [16] (2 genes); PetA, Rieske Iron-Sulfur protein (represented in split columns to indicate the loss of conserved ligands – Fig. S8); PetB, cytochrome *b* (represented in split columns to indicate the loss of conserved ligands for cytochrome b_H, as shown in Supplementary Fig. S1B); Pet C, cytochrome c_1 (Fig. S10); Cyc2, outer membrane cytochrome *c*-porin involved in iron oxido-reduction [70-73] (Fig. 8A); Cyc1, mono- or do-heme cytochrome *c* involved in iron oxido-reduction [70-73]; COXb, COX operon subtype b, mitochondrial-like cytochrome oxidase [20] (7-8 genes); COX A2, A2 type COX operon subtype a-I [20] (10-12 genes); ba3, B-family cytochrome *c* oxidase [20] (4-5 genes); cperox, cytochrome *c* peroxidase (single gene) [14].

	Q	Q reductases										QH2 oxidases			cytochrome bc1							cytochrome oxidases			
**taxon name**	Q	ETF-Q	nuo13	nuo14	Sdh	PQQde	SoxBY	Hyd1	Codh*	**nSor#**	**RC**	bo3	bd-I	Cio bd	PetA		PetB		PetC	Cyc2	Cyc1	COX b	COX A2	ba3	cperox
																	bL	bH							
Elioraea tepidiphila						**3**																			
Rhodospirillales 70-18																									
RickerHillsnord_2_bin.32 clade a																									
RicNunsandstonMG_3_bin.48 clade b						2				2															
SiePeanordMG_2_bin.9 clade b														2											
Acidisphaera sp. L21 LMUY01						2						2		2											
KnobheadnordMG_2_bin.72 LMUY01																							2		
RicNunsandstonMG_4_bin.87 LMUY01										**3**															
Rhodovastum atsumiense	TUV					4		2												2					2
Rhodopila globiformis	TUV					**8**				2	2									**3**					
Acidibrevibacterium fodinaquatile	TUV					**3**															2				
Acidisphaera rubrifaciens						**4**				**3**															
BatPronordMG_2_bin.23 clade c										2															
Acidisoma sp. S159						1						2													2
Acidisoma cellulosilyticum						1								2											
Acidocella sp. MAG 20	TUV					1															**3**				
Rhodospirillales bacterium 20-64-7						2																			
**Acidocella sp. RAIPT20**																									
**Acidocella sp. RAIPT19**								2																	
**Acidocella sp. RAIPG19**						1																			
Acidocella sp. 20-57-95										2															
Acidocella sp. 20-58-15																									
Acidocella sp. 35-58-6																									
Acidocella aminolytica 101						2																			
Acidocella sp. KAb 2-4						2																			
Acidocella sp. MX-AZ02						2						2													
**Acidocella sp. MX-AZ03**						2						2													
Acidocella facilis ATCC 35904						**3**						2		2											
Acidocella aromatica						1																			
Acidiphilium iwatense	TUV					2																			
Acidiphilium rubrum/angustum						2																			
Acidiphilium sp. 21-60-14														2											
RicNunsandstonMG_3_bin.74 clade d										2		2		**3**							**3**				
Lichenicoccus roseus						**4**				2		**3**													
Lichenicola cladoniae						2				**3**		**3**									2				
**Acetobacteraceae sp. KSS8**						1						2													
Acetobacter aceti NBRC 14818						**6**								2											2
Gluconobacter oxydans 621H						**6**																			
Formicincola oecophyllae																									
